# Structural Basis of Metallo-β-Lactamase Inhibition by Captopril Stereoisomers

**DOI:** 10.1128/AAC.01335-15

**Published:** 2015-12-31

**Authors:** Jürgen Brem, Sander S. van Berkel, David Zollman, Sook Y. Lee, Opher Gileadi, Peter J. McHugh, Timothy R. Walsh, Michael A. McDonough, Christopher J. Schofield

**Affiliations:** a Department of Chemistry, University of Oxford, Oxford, United Kingdom; b Structural Genomics Consortium, University of Oxford, Oxford, United Kingdom; c Department of Oncology, Weatherall Institute of Molecular Medicine, University of Oxford, John Radcliffe Hospital, Oxford, United Kingdom; d Department of Microbiology and Infectious Diseases, Institute of Infection and Immunity, Heath Hospital, Cardiff, United Kingdom

## Abstract

β-Lactams are the most successful antibacterials, but their effectiveness is threatened by resistance, most importantly by production of serine- and metallo-β-lactamases (MBLs). MBLs are of increasing concern because they catalyze the hydrolysis of almost all β-lactam antibiotics, including recent-generation carbapenems. Clinically useful serine-β-lactamase inhibitors have been developed, but such inhibitors are not available for MBLs. l-Captopril, which is used to treat hypertension via angiotensin-converting enzyme inhibition, has been reported to inhibit MBLs by chelating the active site zinc ions via its thiol(ate). We report systematic studies on B1 MBL inhibition by all four captopril stereoisomers. High-resolution crystal structures of three MBLs (IMP-1, BcII, and VIM-2) in complex with either the l- or d-captopril stereoisomer reveal correlations between the binding mode and inhibition potency. The results will be useful in the design of MBL inhibitors with the breadth of selectivity required for clinical application against carbapenem-resistant Enterobacteriaceae and other organisms causing MBL-mediated resistant infections.

## INTRODUCTION

The increasing problem of antibiotic resistance is a global health concern (1), with the World Health Organization (WHO) and the European Centre for Disease Prevention and Control (ECDPC) reporting that several million people are infected with antibiotic-resistant bacteria annually. It is estimated that >50,000 patients die each year due to infections caused by multidrug-resistant bacterial pathogens in the United States alone (2).

β-Lactam-containing compounds remain the most important antibiotics in clinical use, but their effectiveness is threatened by increasing resistance. β-Lactam resistance is most importantly mediated by serine- and zinc-dependent metallo-β-lactamases (SBLs and MBLs, respectively), which catalyze β-lactam hydrolysis (3). In combination with an appropriate penicillin antibiotic, class A SBL (penicillinase) inhibitors (i.e., clavulanic acid, tazobactam, and sulbactam) have been used widely in the clinic, and recently, a class C (cephalosporinase) SBL inhibitor (4), Avibactam, was approved for clinical use in combination with a cephalosporin (5). In contrast, there are no reports of clinically useful MBL inhibitors (6).

**FIG 1:**
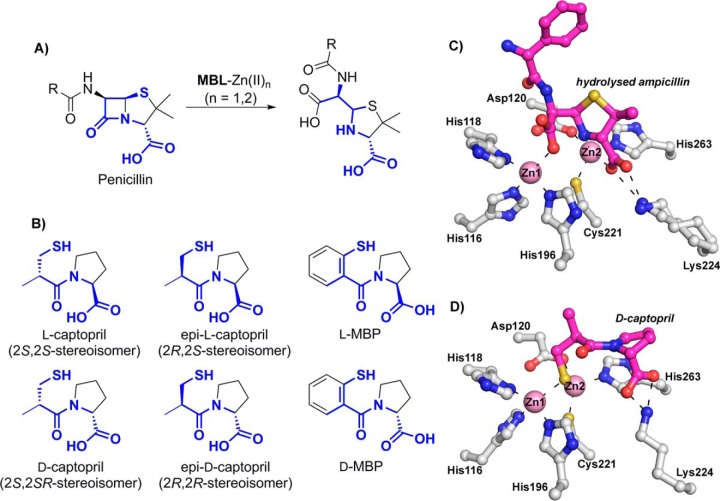
Captopril has structural similarity to the hydrolyzed penicillin product of metallo-β-lactamase catalysis (penicillinoic acid). (A) Outline of the mode of action of metallo-β-lactamases (MBLs). (B) Structures of the four captopril stereoisomers (2*S*,2*S*, 2*S*,2*R*, 2*R*,2*S*, and 2*R*,2*R*) and of d- and l-MBP. (C) Binding mode of hydrolyzed ampicillin with NDM-1 (PDB code 4HL2). (D) Binding mode of d-captopril with IMP-1 (PDB code 4C1G [described in this study]).

A challenge for the development of useful MBL inhibitors is achieving a breadth of inhibition against most MBL subtypes while avoiding inhibition of structurally related human MBL-fold enzymes (7). Crystal structures reveal that MBLs have a characteristic αβ/βα sandwich fold, that they possess conserved zinc ion binding sites, and that loops flanking the active site are involved in ligand binding (8). MBLs can be divided into three subclasses (B1, B2, and B3), based on the number of zinc ions in their metal binding sites and/or based on sequence and structural similarities (6). B1 MBLs (e.g., imipenemase [IMP], Verona integron-encoded MBL [VIM], and New Delhi MBL [NDM] types) are the most clinically relevant MBLs; these MBLs catalyze hydrolysis of almost all β-lactams, including the latest generations of cephalosporins and carbapenems (9). Several classes of known metalloenzyme inhibitors inhibit MBLs, including thiols, carboxylic acids, trifluoromethyl ketones, hydroxamic acids, and rhodanines (7, 10, 11) (see Fig. S1 in the supplemental material for structures).

(2*S*)-1-[(2*S*)-2-Methyl-3-sulfanylpropanoyl] pyrrolidine-2-carboxylic acid (commonly referred to as l-captopril) (Fig. 1) is a thiol-containing small molecule which was developed in the 1970s to target the zinc ion-utilizing human angiotensin-converting enzyme (ACE) (12, 13). l-Captopril was used successfully for several decades to control high blood pressure. The clinically used (2*S*,2*S*)-stereoisomer, i.e., l-captopril, inhibits several MBLs from all subclasses (14–18). However, the (2*S*,2*R*)-stereoisomer, (2*S*)-1-[(2*R*)-2-methyl-3-sulfanylpropanoyl] pyrrolidine-2-carboxylic acid (Fig. 1), commonly referred to as d-captopril, has been reported to be more active than l-captopril against some MBLs (e.g., NDM-1 [19], BcII [17], CcrA [20], and CphA [17]).

**FIG 2:**
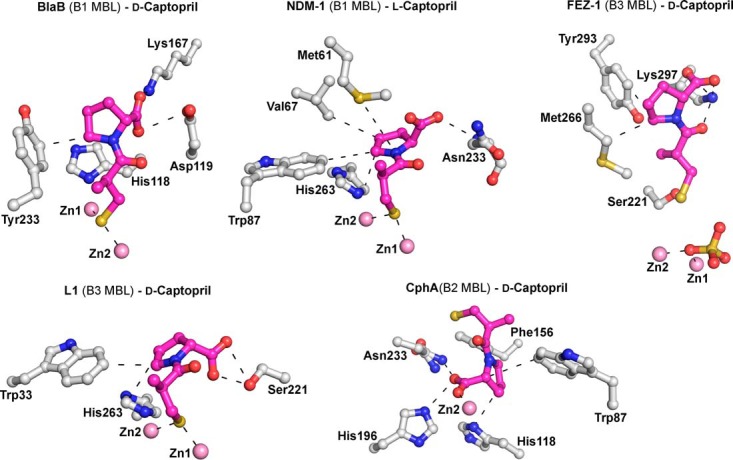
Crystallographic analysis reveals different binding modes for d- and l-captopril. Preliminary crystal structures are shown for BlaB, NDM-1, CphA, FEZ-1, and L1 complexed with l- and d-captopril (PDB entries 1M2X [1.50 Å], 4EXS [2.40 Å], 2QDS [1.66 Å], 1JT1 [1.65 Å], and 2FU8 [1.80 Å]). Zinc ions are represented by pink spheres, d- and l-captopril ligands are shown in magenta, and the amino acid residues interacting with captopril are depicted as gray stick models. Hydrogen bonds, zinc coordination bonds, and hydrophobic interactions are shown as thin black dashes.

Crystal structures have been reported for some MBLs in complex with l- or d-captopril, i.e., (i) in the case of the B1 subclass MBLs, for NDM-1 complexed with l-captopril (21) and for Chryseobacterium meningosepticum BlaB complexed with d-captopril (22); (ii) in the case of the B2 MBLs, for Aeromonas hydrophila CphA complexed with d-captopril (18); and (iii) in the case of the B3 MBLs, for the Fluoribacter gormanii MBL FEZ-1 complexed with d-captopril (23) and the Stenotrophomonas maltophilia MBL L1 complexed with d-captopril (15). Biophysical analyses employing extended X-ray absorption fine structure (EXAFS) and perturbed angular correlation of X-rays (PAC) spectroscopy have been reported for BcII and CphA complexed with d- and l-captopril (17). Molecular dynamic calculations on d- and l-captopril complexed with BcII and d-captopril complexed with NDM-1 have also been reported (20, 24). These analyses imply that both l- and d-captopril can bind with the thiol(ate) ligated to both active site Zn(II) ions (Fig. 2; see Fig. S2 to S4 in the supplemental material). Interestingly, despite BlaB and NDM-1 belonging to the same B1 MBL subclass, different binding modes were observed for the l- and d-captopril stereoisomers (19). In the case of the mono-Zn(II) ion-binding B2 subclass, a structure of the CphA–d-captopril complex (18) indicates that the d-captopril carboxylate, rather than the thiol(ate), binds to the single Zn(II) ion, a binding mode that possibly reflects the relatively weak inhibition of this enzyme by d-captopril (*K_i_* = 72 μM). Finally, for the B3 MBL subclass, in a crystal structure of the FEZ-1–d-captopril complex (23), the binding of captopril was modeled such that neither the d-captopril thiol nor its carboxylate interacts with the active site Zn(II) ions, a binding mode that was also proposed to be consistent with the relatively weak inhibition observed in this case (*K_i_* = 400 μM) (see Fig. S2 in the supplemental material). To date, there have been no reports on MBL inhibition by (2*R*)-1-[(2*S*)-2-methyl-3-sulfanylpropanoyl] pyrrolidine-2-carboxylic acid, subsequently referred to as *epi*-l-captopril (the 2*R*,2*S*-stereoisomer), or (2*R*)-1-[(2*R*)-2-methyl-3-sulfanylpropanoyl] pyrrolidine-2-carboxylic acid, subsequently referred to as *epi*-d-captopril (the 2*R*,2*R*-stereoisomer) (Fig. 1).

We report systematic studies on the inhibition of four clinically relevant MBLs (IMP-1, VIM-2, SPM-1, and NDM-1) and the model MBL BcII by the four captopril stereoisomers and both enantiomers of a captopril derivative, 1-(2-mercaptobenzoyl)pyrrolidine-2-carboxylic acid (d- and l-MBP) (Fig. 1; see Table S1 in the supplemental material) (25, 26). The combined kinetic and structural studies clearly reveal different binding modes for different captopril stereoisomers and will help to enable the future development of broad-spectrum MBL inhibitors.

## MATERIALS AND METHODS

### Synthesis.

The different captopril isomers and captopril derivatives were prepared according to procedures in the literature (see Schemes S1 and S2 and the experimental section in the supplemental material).

### Protein production and purification.

Recombinant forms of NDM-1, VIM-2, VIM-4, SPM-1, IMP-1, and BcII MBLs were produced in Escherichia coli as described previously (27, 28). Purified proteins were dialyzed into freshly prepared crystallization buffer (50 mM HEPES, pH 7.5, 150 mM NaCl containing 1 μg ZnCl_2_) and then concentrated (to 2 mM [BcII], 0.75 mM [IMP-1], and 0.36 mM [VIM-2]) before use in crystallization studies.

### Crystallography.

Crystals were grown under the conditions stated in Table S2 in the supplemental material and were cryoprotected using well solution diluted with 25% glycerol before being flash cooled in liquid nitrogen. All data sets were collected at 100 K. All data were indexed, integrated, and scaled using HKL-3000 (29). The structures were solved by molecular replacement using Phaser (30). The structures were then refined using PHENIX (31) and COOT (32) until the *R*_work_ and *R*_free_ values no longer decreased. Data collection and refinement statistics are given in Tables S3 to S5 in the supplemental material.

### Kinetic analyses.

Kinetic and inhibition assays with the bacterial MBLs, human angiotensin-converting enzyme 2 (hACE-2), and hydroxyacylglutathione hydrolase, human glyoxylase II (hHAGH), were performed as described previously (7, 27).

### Nuclease assays.

Nuclease assays with DCLRE1A and DCLRE1B (DNA cross-link repair enzymes 1A and 1B) (33) were performed according to a previously described method (33), employing a 21-nucleotide DNA oligonucleotide with a fluorescein label at its 3′ end. In brief, exonuclease activity was measured using ΔN-DCLRE1A (3.5 ng; 8 nM) or ΔC-DCLRE1B (1.5 ng; 4 nM) mixed with 1 pmol (1 μM) of 3′-fluorescein-labeled DNA substrate in 10 μl of 20 mM HEPES, pH 7.9, 50 mM KCl, 10 mM MgCl_2_, 0.5 mM dithiothreitol (DTT), 0.05% Triton-X, 0.1 mg/ml bovine serum albumin (BSA), and 5% glycerol. Reaction mixtures were incubated at 37°C for 20 min with the indicated concentrations of d-captopril, l-captopril, d-MBP, or l-MBP (see Fig. S5 in the supplemental material), and reactions were then quenched by addition of 2 μl of 80% formamide-10 mM EDTA and heating at 95°C for 5 min. Following separation in a 20% polyacrylamide-7 M urea denaturing gel, substrate and product bands were visualized by use of a Typhoon Trio+ variable-model imager (excitation at 488 nm with a blue laser at 400 V).

### MIC determinations.

The bacteria used were nonclonal international isolates whose mechanisms of carbapenem resistance have been defined genetically by sequencing. E. coli ATCC 25922 was used as a negative control. MBL genes were obtained by PCR using standard procedures and were inserted into a pK18 vector which was used to transform E. coli J53 (34). Transconjugants were obtained with MBL genes carried on native wild-type plasmids conjugated into J53 (35). Both transformants and transconjugants were confirmed by DNA sequencing and verified for MBL production by using MTS MBL strips (Liofilchem, Roseto, Italy). Additionally, 5 Escherichia coli (with NDM-1), 10 Klebsiella pneumoniae (5 with NDM-1, 3 with VIM-4, and 2 with IMP-4), 1 Serratia marcescens (with IMP-4), and 2 Pseudomonas aeruginosa (with VIM-2 and AIM-1) isolates and E. coli ATCC 25922 were used in the test panel. MICs were determined using the broth microdilution method according to CLSI guidelines. Strains were cultured and tested in cation-adjusted Mueller-Hinton agar and broth (Becton Dickinson).

### Protein structure accession numbers.

Coordinates and structure factors have been deposited in the Protein Data Bank (PDB) under the following accession codes: for apo di-Zn(II)–BcII, PDB entry 4C09; for BcII–l-captopril, PDB entry 4C1H; for BcII–d-captopril, PDB entry 4C1C; for IMP-1–l-captopril, PDB entry 4C1F; for IMP-1–d-captopril, PDB entry 4C1G; for apo di-Zn(II)–VIM-2, PDB entry 4BZ3; for VIM-2–l-captopril, PDB entry 4C1D; and for VIM-2–d-captopril, PDB entry 4C1E. The URL for coordinate deposition is http://rcsb-deposit.rutgers.edu/.

## RESULTS

### MBL inhibition by captopril stereoisomers.

**FIG 3:**
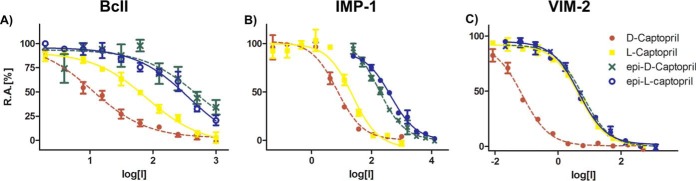
IC_50_ curves for all captopril stereoisomers tested against BcII (A), IMP-1 (B), and VIM-2 (C) reveal different potencies. The l- and *epi*-l-captopril stereoisomers are represented by solid lines, and the d- and *epi*-d-captopril stereoisomers are depicted with dashed lines. R.A., remaining activity.

We first synthesized the four possible captopril stereoisomers (d-, l-, *epi*-d-, and *epi*-l-captopril) (see Schemes S1 and S2 and the experimental section in the supplemental material) and tested them as inhibitors against BcII and clinically relevant MBLs from the B1 subclass (IMP-1, VIM-2, SPM-1, and NDM-1) (Table 1 and Fig. 3) (9). Comparing the previously reported d-captopril and l-captopril inhibition values to our results for NDM-1, IMP-1, and BcII reveals relatively small differences (Table 1), likely due at least in part to different assay conditions (for d-captopril, NDM-1 IC_50_s of 20.1 μM versus 7.9 μM [36] and BcII IC_50_s of 10.7 μM versus 45 μM [*K_i_*] [17]; and for l-captopril, NDM-1 IC_50_s of 157.4 μM versus 202 μM [36], IMP-1 IC_50_s of 7.2 μM versus 12.5 μM [*K_i_*] [37], and BcII IC_50_s of 80.4 μM versus 65 μM [*K_i_*] [17]). In all cases, d-captopril was the most potent of the four possible captopril stereoisomers (NDM-1 IC_50_, 20.1 ± 1.5 μM; IMP-1 IC_50_, 7.2 ± 1.2 μM; VIM-2 IC_50_, 0.072 ± 0.010 μM; SPM-1 IC_50_, 261.8 ± 1.3 μM; and BcII IC_50_, 10.7 ± 1.2 μM) (Table 1). d-Captopril was consistently more potent than l-captopril (∼7-fold for NDM-1, ∼3-fold for IMP-1, ∼60-fold for VIM-2, ∼2-fold for SPM-1, and ∼8-fold for BcII) (Table 1). Both the *epi*-l- and *epi*-d-captopril stereoisomers were poor inhibitors of BcII and SPM-1 (IC_50_s were all ≥500 μM), whereas for NDM-1 and IMP-1, unlike *epi*-l-captopril, *epi*-d-captopril showed some activity (NDM-1 IC_50_, 64 μM; and IMP-1 IC_50_, 173 μM). Relatively potent IC_50_s were observed for both *epi*-l- and *epi*-d-captopril against VIM-2 (IC_50_ = 5.5 μM). The captopril derivatives d- and l-MBP were less potent (IC_50_s of >500 μM) than d- and l-captopril against all MBLs (Table 1).

**TABLE 1:** IC_50_s for the four captopril stereoisomers and derivatives of captopril (MBP) against different MBLs

Compound	IC_50_ (μM)^*c*^				
	BcII	IMP-1	VIM-2	SPM-1	NDM-1
d-Captopril	10.7 ± 1.2 (45^*a*^)	7.2 ± 1.2	0.072 ± 0.01	261.8 ± 1.3	20.1 ± 1.5 (7.9^*b*^)
l-Captopril	80.4 ± 1.1 (65^*a*^)	23.3 ± 1.3(12.5^*a*^)	4.4 ± 0.8	>500	157.4 ± 1.3 (202^*b*^)
*epi*-d-Captopril	>500	173.2 ± 1.2	5.5 ± 0.7	>500	64.6 ± 1.4
*epi*-l-Captopril	423.8 ± 1.5	436 ± 1.1	5.5 ± 1.5	>500	>500
d-MBP	>500	>500	>500	>500	>500
l-MBP	>500	>500	>500	>500	>500

a *K_i_* value from the literature.

b IC_50_ from the literature.

c All experiments were performed three or more times. Nonlinear regression analysis was used to calculate the IC_50_s and their corresponding 95% confidence intervals (GraphPad Prism). Results represent means ± standard deviations.

A key issue in work toward obtaining clinically relevant MBL inhibitors is the degree of selectivity toward the bacterial MBLs over human metalloenzymes, including MBL-fold enzymes. Although l-captopril is a well-studied ACE-2 inhibitor, there are no reports of its selectivity versus human MBL-fold enzymes. We tested l- and d-captopril as well as d- and l-MBP against the human MBL-fold enzymes DCLRE1A and DCLRE1B (33), hHAGH, and hACE-2 (zinc-dependent human metalloenzyme); no inhibition was observed at 100 μM under our standard assay conditions (see Fig. S5 in the supplemental material).

### Pathogen susceptibility to d- and l-captopril.

Since the d- and l-captopril isomers were consistently the most potent stereoisomers against the tested MBLs (Table 2), we tested them against nonclonal multidrug-resistant bacteria expressing various MBLs. We used a variety of isolates with cloned MBLs, transconjugants, and wild-type clinical isolates. The panel of strains was tested for meropenem and ceftazidime MICs, with and without l- or d-captopril at 8 mg/liter (Table 2). In order to correlate the data with the results of cellular studies (see below), we determined the IC_50_s for d- and l-captopril against VIM-4 (VIM-4 IC_50_ of 1.7 ± 0.4 μM for d-captopril and 3.9 ± 0.5 μM for l-captopril). Pathogenic strains with different geographical origins (e.g., Greece [A-33; VIM-4] or India [IR60; NDM-1]) (38) and displaying resistance to β-lactams as well as fluoroquinolone and aminoglycoside antibiotics were selected for MIC tests (Table 2). While l-captopril showed potentiation with cloned and transconjugated MBLs, there was little synergy observed against wild-type strains, and it was generally less than that with d-captopril (Table 2). The addition of d-captopril potentiated the efficacy of meropenem against most of the VIM-2-, VIM-4-, IMP-4-, and NDM-1-producing strains tested, including E. coli, K. pneumoniae, S. marcescens, and P. aeruginosa (Table 2).

**TABLE 2:** MICs of meropenem (MEM) and ceftazidime (CFZ), with and without d- and l-captopril (d- and l-CAP), versus various Gram-negative bacteria

Strain (inhibitor)	Genotype	MIC					
		MEM	MEM + d-CAP (8 mg/liter)	MEM + l-CAP (8 mg/liter)	CFZ	CFZ + d-CAP (8 mg/liter)	CFZ + l-CAP (8 mg/liter)
E. coli 25922		<0.125	<0.125	<0.125	0.125	<0.125	<0.125
E. coli J53 + NDM-1 clone	*bla*_NDM-1_	64	8	8	512	8	32
E. coli J53 + NDM-1 transconjugant	*bla*_NDM-1_	128	16	16	512	16	32
E. coli J53 + VIM-2 clone	*bla*_VIM-2_	8	2	4	32	8	16
E. coli J53 + VIM-2 transconjugant	*bla*_VIM-2_	2	1	1	32	4	8
E. coli J53 + IMP-1 clone	*bla*_IMP-1_	1	0.25	0.5	128	32	32
K. pneumoniae IR16 (NDM-1)	*bla*_DHA-1_ *bla*_CTX-15_ *bla*_TEM-1_ *bla*_SHV-1_ *bla*_NDM-1_	128	8	128			
E. coli IR10 (NDM-1)	*bla*_DHA-1_ *bla*_CTX-15_ *bla*_TEM-1_ *bla*_OXA-1_ *bla*_NDM-1_	64	2	64			
K. pneumoniae IR8 (NDM-1)	*bla*_DHA-1_ *bla*_CTX-15_ *bla*_TEM-1_ *bla*_SHV-1_ *bla*_NDM-1_	16	2	16			
E. coli IR15 (NDM-1)	*bla*_DHA-1_ *bla*_CTX-15_ *bla*_TEM-1_ *bla*_OXA-1_ *bla*_NDM-1_	8	0.25	8			
K. pneumoniae IR19 (NDM-1)	*bla*_DHA-1_ *bla*_CTX-15_ *bla*_TEM-1_ *bla*_SHV-1_ *bla*_NDM-1_	8	1	8			
E. coli IR24 (NDM-1)	*bla*_DHA-1_ *bla*_CTX-15_ *bla*_TEM-1_ *bla*_OXA-1_ *bla*_NDM-1_	512	32	512			
E. coli IR60 (NDM-1)	*bla*_DHA-1_ *bla*_CTX-15_ *bla*_TEM-1_ *bla*_OXA-1_ *bla*_NDM-1_	128	32	128			
K. pneumoniae HR8 (NDM-1)	*bla*_DHA-1_ *bla*_CTX-15_ *bla*_TEM-1_ *bla*_OXA-1_ *bla*_SHV-1_ *bla*_NDM-1_	64	4	64			
K. pneumoniae N16 (NDM-1)	*bla*_DHA-1_ *bla*_CTX-15_ *bla*_TEM-1_ *bla*_SHV-1_ *bla*_NDM-1_	32	4	16			
K. pneumoniae A33 (VIM-4)	*bla*_CTX-15_ *bla*_TEM-1_ *bla*_SHV-12a_ *bla*_VIM-4_	8	1	16			
K. pneumoniae A34 (VIM-4)	*bla*_CTX-15_ *bla*_TEM-1_ *bla*_SHV-12a_ *bla*_VIM-4_	8	1	8			
K. pneumoniae A35 (VIM-4)	*bla*_CTX-15_ *bla*_TEM-1_ *bla*_SHV-12a_ *bla*_VIM-4_	16	4	16			
K. pneumoniae B12 (IMP-4)	*bla*_CTX-15_ *bla*_TEM-1_ *bla*_SHV-12a_ *bla*_IMP-4_ *bla*_OXA-1_	8	1	4			
S. marcescens B13 (IMP-4)	*bla*_DHA-1_ *bla*_AMPC_ *bla*_TEM-1_ *bla*_IMP-4_	4	1	4			
K. pneumoniae B19 (IMP-4)	*bla*_CTX-15_ *bla*_TEM-1_ *bla*_SHV-12a_ *bla*_IMP-4_	32	2	16			
P. aeruginosa 4470 (VIM-2)	*bla*_AMPC_ *bla*_VIM-2_	512	512	512			
P. aeruginosa (AIM-1)	*bla*_AMPC_ *bla*_AIM-1_	512	512	512			

### Structural analysis of captopril binding to IMP-1, VIM-2, and BcII.

We next investigated the mode of binding of the captopril stereoisomers to MBLs by using crystallography. We determined high-resolution crystal structures for d- and l-captopril in complex with IMP-1 (1.71- and 2.01-Å resolutions, respectively), VIM-2 (1.40- and 1.20-Å resolutions, respectively), and BcII (1.18- and 1.10-Å resolutions, respectively). For comparison, structures of di-Zn(II)–VIM-2 and di-Zn(II)–BcII without inhibitors were also determined, to 1.20- and 1.30-Å resolutions, respectively. For all structures, the crystal systems were similar to those reported previously (for VIM-2 [7], IMP-1 [39], and BcII [40]). (Note that we used the standard numbering scheme for class B β-lactamases, i.e., BBL numbering [41].)

As anticipated, in all cases the overall protein folds observed were characteristic αβ/βα MBL sandwich folds (2 β-sheets sandwiched with 2 helices buttressed against each external face of the sandwich) (8). The active sites, which are located at one end of the two β-sheets in a groove surrounded by several loops, were occupied in all cases by two zinc ions, as expected for B1 subclass MBLs. The L3 and L10 loops, which flank the active site, are located opposite each other and are involved in substrate binding (42). The L3 loop (residues 61 to 66 [BBL numbering]) is located between strands β3 and β4, and the L10 loop (residues 223 to 241) is located between strand β11 and helix α4, which includes Lys224/Arg228 and Asn233, whose side chains are directly involved in substrate and inhibitor binding (41, 43).

The electron density maps for the ligands in the various MBL-captopril complexes suggested various ligand occupancies and were carefully analyzed between rounds of refinement (see Fig. S6 in the supplemental material). For BcII, both the d- and l-captopril isomers were modeled and refined with 70% occupancy. For the VIM-2–d-captopril complex structure, the ligands were modeled and refined with 100% occupancy, and for the VIM-2–l-captopril complex structure, 80% occupancy was used. For the IMP-1–d-captopril and IMP-1–l-captopril structures, the ligands were modeled and refined with 100% occupancy in chain A, but the residual density present in chain B was interpreted as too weak (<50% occupancy) to include in the model.

Preliminary structural analysis indicated that partial oxidation of the metal-binding cysteine (Cys221) had occurred in both the crystallized BcII and VIM-2 proteins, in a manner similar to that observed in previous crystallographic studies of these B1 MBLs (40, 44). Due to the likelihood of active site cysteine oxidation (Cys221) interfering with the active site Zn(II) chemistry, and hence with our analysis of ligand binding, we worked to minimize Cys221 oxidation. In all cases, cysteine oxidation during crystallization could be prevented by the addition of tris(2-carboxyethyl)phosphine (TCEP) (40) (except for IMP-1, for which cysteine oxidation in the absence of TCEP was not observed).

Our MBL-captopril complex structures show an overall captopril binding mode similar to those previously observed for NDM-1–l-captopril (21), BlaB–d-captopril (22), and L1–d-captopril (15), but they differ significantly from the FEZ-1–d-captopril (23) and CphA–d-captopril complex structures (Fig. 3; see Fig. S2 in the supplemental material). Our MBL–l-captopril structures are most similar to the reported NDM-1–l-captopril complex structure (21), and our MBL–d-captopril structures are most similar to the L1–d-captopril complex structure (15) (see Fig. S2 in the supplemental material). Comparison of the MBL-captopril complex structures with the active site of apo-MBLs reveals that several water molecules are displaced upon binding of d- or l-captopril (see Fig. S7 in the supplemental material); these displacements likely contribute to the strength of inhibitor binding.

**FIG 4:**
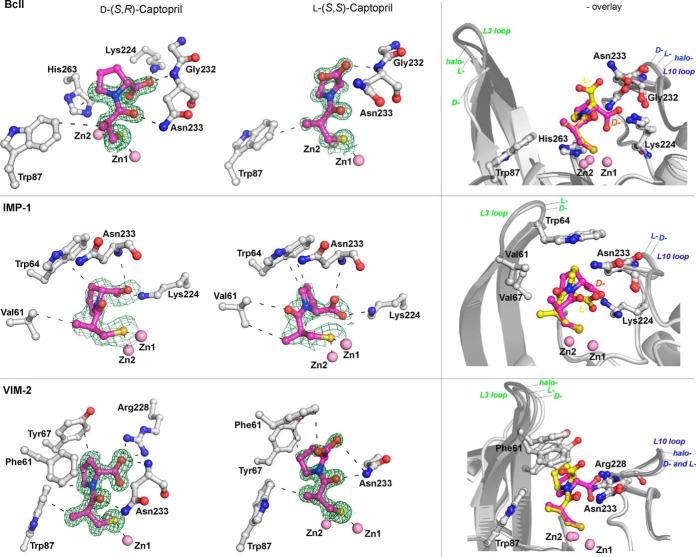
Crystallographic analyses reveal different binding modes for d- and l-captopril. The left column shows views of structures of BcII, IMP-1, and VIM-2 complexed with l- and d-captopril (PDB entries 4C1H [1.10 Å], 4C1C [1.18 Å], 4C1F [2.01 Å], 4C1G [1.71 Å], 4C1D [1.20 Å], and 4C1E [1.40 Å], respectively), highlighting residues involved in inhibitor-MBL complex formation. The right column shows an overlay of structures in the absence/presence of d- or l-captopril; these reveal L3 and L10 loop movements on inhibitor binding. With BcII, a comparison of the L3 loop was not possible, because some part of it was not modeled, but clear movement was identified for the L10 loop. In the case of IMP-1, we did not obtain a di-Zn(II) structure without an inhibitor; a comparison with published IMP-1 structures is difficult because of different crystallization conditions, but in the d- and l-captopril structures both the L3 and L10 loops display different conformations. Zinc atoms are represented by pink spheres, the d- and l-captopril ligands are shown in magenta, and the amino acid residues interacting with captopril are shown as gray stick models. The electron density maps (F_o_-F_c_) are contoured to 3.0 σ and shown in green. Hydrogen bonds, zinc coordination bonds, and hydrophobic interactions are shown with thin black dashes. The MBL backbone in the overlay plots is shown in gray, and the flexible active site loops are shown in different shades of gray (loops L3 and L10).

Captopril has distinct features that enable metalloprotein binding. The thiol acts as a metal binding ligand that displaces the proposed “hydrolytic” water molecule (or hydroxide) that bridges the two active site metal ions. An amide carbonyl group leads to the conformationally constrained prolyl ring, and a methyl group extends from the carbon bonded to the thiol. Both the l- and d-captopril diastereoisomers present two distinct binding faces (Fig. 4). One face is hydrophobic and is formed by the methyl group and the proline ring methylenes; the hydrophobic face interacts with residues from the L3 loop (Trp87_BcII_; Trp64_IMP-1_ and Val61_IMP-1_; and Trp87_VIM-2_, Phe61_VIM-2_, and Tyr67_VIM-2_) (Fig. 4). Such interactions are in agreement with the proposed role of the “mobile” L3 loop in interacting with the hydrophobic *N*-acyl substituents of cephalosporin and penicillin MBL substrates (45). The other face of captopril is more hydrophilic and is positioned to form hydrogen bonds to residues in the L10 loop. In all our MBL–d-captopril structures, the d-captopril carboxylate is positioned to form electrostatic and hydrogen-bonding interaction distances ranging from 2.3 Å for IMP-1 to 2.9 Å for BcII and VIM-2, with a conserved positively charged residue (Lys224_IMP-1,BcII_ or Arg228_VIM-2_) which is predicted to be involved in binding the β-lactam substrate carboxylate (46, 47) (Fig. 4; Fig. S4 in the supplemental material). As observed for our MBL–d-captopril structures, the IMP-1–l-captopril structure has the inhibitor carboxylate positioned to form an electrostatic interaction with a conserved basic residue, i.e., Lys224. In contrast, in the BcII–l-captopril and VIM-2–l-captopril structures, the captopril carboxylate is oriented away from the conserved positively charged Lys/Arg residue (Lys224/Arg228). In all MBL–l-captopril structures (including those with IMP-1), the carboxylate is positioned to interact with the conserved asparagine (Asn233) from the L10 loop. IMP-1 is thus apparently a special case, i.e., both the l- and d-captopril isomers bind in similar modes with the inhibitor carboxylate positioned to interact with Lys224_IMP-1_. In the structures of BcII and VIM-2 complexed with d-captopril and VIM-2 complexed with l-captopril, the captopril amide carbonyl oxygen is positioned to interact with the conserved asparagine (Asn233) from the L10 loop (Fig. 4; see Fig. S4 in the supplemental material). In all cases, the L3 and L10 loops were observed to move slightly toward the inhibitors relative to their positions in the absence of inhibitor, consistent with an induced-fit mechanism during substrate binding. This is most clearly observed in the case of VIM-2 (Fig. 4).

The observed binding modes for d-captopril carboxylate in our structures differ from the binding mode observed in the reported BlaB–d-captopril structure (22), despite a similar binding mode for the captopril thiol(ate) binding to the zinc ions. In the case of BlaB, the d-captopril carboxylate is rotated ∼180° relative to the thiolate mode of binding in our structures, such that it does not interact with the conserved Lys224_BlaB_ but binds to Lys167_BlaB_ from the L10 loop. The conserved Asn233_BBL_ in the L10 loop is replaced by tyrosine in BlaB (22); this Asn-Tyr substitution likely contributes to the different d-captopril binding mode in BlaB compared to that in our structures. Thus, d-captopril is likely to bind to NDM-1, which contains an asparagine (Asn233_BBL_) rather than a tyrosine at this position, in a manner similar to that observed for BcII, IMP-1, and VIM-2 (rather than the BlaB binding mode) (21).

In all of our MBL-captopril structures, the distance from the Zn(II) atoms to the bridging thiolate sulfur atom is ∼2.3 Å. Similar distances have been reported for the BlaB (B1)– and L1 (B3)–d-captopril structures (PDB entries 1M2X and 2FU8), with a slightly shorter distance for the l-captopril–NDM-1 structure (PDB entry 4EXS) (21). Different values have been observed for the MBL intermetal distance in the absence of exogenous ligands, with reported values ranging from 2.5 to 4.5 Å (48); this may be dependent in part on the actual metals bound, which is not always possible to assign based on diffraction data alone (48). An interzinc distance of 3.5 Å was observed for both our di-Zn(II)–BcII and di-Zn(II)–VIM-2 apo structures, in which a bridging water was present as previously observed. The inter-zinc distances increased to 3.8 Å and 3.7 Å upon d- or l-captopril binding to BcII and VIM-2, respectively, with the binding thiolate. This observation is consistent with the different van der Waals atomic radii for sulfur (1.8 Å) and oxygen (1.5 Å). For IMP-1, the observed Zn-Zn distance (3.5 Å [PDB entry 1DDK] [39], as in our BcII and VIM-2 structures) increased to 3.7 Å on binding of either the l- or d-captopril stereoisomer.

## DISCUSSION

Although once of little clinical relevance, MBLs now have increasing importance (6). The VIM-type B1 MBLs are a major problem in parts of Asia, being present in up to 99% of MBL-positive multidrug-resistant strains (49). Thus, there is a genuine need for a response to MBL-mediated resistance. The finding that d-captopril, a stereoisomer of the clinically used drug l-captopril, is consistently the most potent inhibitor among the captopril stereoisomers against MBLs and can potentiate the effects of meropenem against VIM-2- and other MBL-expressing pathogens is interesting.

Although several crystal structures of MBLs in combination with either d- or l-captopril have been reported, structures of the same MBL in complex with the d- and l-captopril isomers have not been reported previously. The captopril isomer binding modes that we observed are related in that they all involve thiol(ate) zinc chelation, as in most of the previously reported structures. An exception is the reported FEZ-1–d-captopril structure (23), which may not be representative of binding in solution due to its relatively poor quality.

Correlations can be made between the observed binding modes and the potency of inhibition. More potent inhibition was always observed when an interaction between the captopril carboxylate and the conserved basic Lys/Arg (Lys224_BBL_) involved in substrate binding was shown in the crystal structures. In all cases, VIM-2 manifested lower IC_50_s than those of the other tested MBLs—this correlates with the additional interactions observed between Arg228_VIM-2_ and the captopril carboxylate, as well as with additional interactions of the VIM-2 L3 loop with the hydrophobic face of captopril. Second, the observation of a decreased number of hydrogen bonding/electrostatic interactions for l- over d-captopril generally reflects weaker inhibition. l-Captopril was observed to be more potent against IMP-1 than against the other MBLs tested, and its IC_50_ was only 3-fold higher than that of d-captopril. This observation of relatively potent inhibition of IMP-1 by l-captopril correlates with the observation that for IMP-1, but none of the other MBLs, the l-captopril carboxylate favors binding to Lys224_IMP-1_.

Product inhibition is commonly observed for MBLs (16). Comparison of our MBL-captopril structural complexes with MBL–β-lactam product complexes (PDB entry 4HL2) (Fig. 1) shows that the most potent of the captopril isomers, d-captopril, has a mode of binding most similar to that of hydrolyzed β-lactams (50), especially penicillins, consistent with d-captopril being the most potent inhibitor (Table 1). However, d-captopril was significantly less potent (>20-fold) against SPM-1 than against all other MBLs tested; the other captopril stereoisomers did not inhibit SPM-1 (IC_50_s of >500 μM). This difference may reflect the unusual nature of SPM-1, as a proposed B1/B2 hybrid MBL (42). A challenge in MBL inhibition is to obtain breadth of selectivity toward the majority of prokaryotic MBLs, which often have relatively low sequence similarity (∼30% for the MBLs we used) (see Table S1 in the supplemental material), without inhibiting the related human MBL-fold enzymes. The combined structural and inhibition results reveal that captopril stereoisomers can potently inhibit B1 MBLs via related but sometimes different binding modes. These observations may be important in developing potent inhibitors with the required breadth of selectivity against different subtypes of MBLs, i.e., medicinal chemists may specifically aim to identify single compounds that bind differently to different MBL subtypes.

## Supplementary Material

Supplemental material

## References

[1] Berendonk TU, Manaia CM, Merlin C, Fatta-Kassinos D, Cytryn E, Walsh F, Burgmann H, Sorum H, Norstrom M, Pons M-N, Kreuzinger N, Huovinen P, Stefani S, Schwartz T, Kisand V, Baquero F, Martinez JL 2015 Tackling antibiotic resistance: the environmental framework. Nat Rev Microbiol 13:310–317. doi:10.1038/nrmicro3439. 25817583

[2] 2013 The antibiotic alarm. Nature 495:141. 10.1038/495141a 23495392

[3] Bush K 2013 Proliferation and significance of clinically relevant β-lactamases. Ann N Y Acad Sci 1277:84–90. doi:10.1111/nyas.12023. 23346859

[4] Bebrone C, Lassaux P, Vercheval L, Sohier JS, Jehaes A, Sauvage E, Galleni M 2010 Current challenges in antimicrobial chemotherapy: focus on ss-lactamase inhibition. Drugs 70:651–679. doi:10.2165/11318430-000000000-00000. 20394454

[5] Ehmann DE, Jahić H, Ross PL, Gu RF, Hu J, Durand-Réville TF, Lahiri S, Thresher J, Livchak S, Gao N, Palmer T, Walkup GK, Fisher SL 2013 Kinetics of avibactam inhibition against class A, C, and D β-lactamases. J Biol Chem 288:27960–27971. doi:10.1074/jbc.M113.485979. 23913691 PMC3784710

[6] Cornaglia G, Giamarellou H, Rossolini GM 2011 Metallo-β-lactamases: a last frontier for β-lactams? Lancet Infect Dis 11:381–393. doi:10.1016/S1473-3099(11)70056-1. 21530894

[7] Brem J, van Berkel SS, Aik W, Rydzik AM, Avison MB, Pettinati I, Umland KD, Kawamura A, Spencer J, Claridge TD, McDonough MA, Schofield CJ 2014 Rhodanine hydrolysis leads to potent thioenolate mediated metallo-β-lactamase inhibition. Nat Chem 6:1084–1090. doi:10.1038/nchem.2110. 25411887

[8] Carfi A, Pares S, Duee E, Galleni M, Duez C, Frère JM, Dideberg O 1995 The 3-D structure of a zinc metallo-β-lactamase from Bacillus cereus reveals a new type of protein fold. EMBO J 14:4914–4921. 7588620 10.1002/j.1460-2075.1995.tb00174.x PMC394593

[9] Karsisiotis AI, Damblon CF, Roberts GCK 2014 A variety of roles for versatile zinc in metallo-β-lactamases. Metallomics 6:1181–1197. doi:10.1039/c4mt00066h. 24696003

[10] Fast W, Sutton LD 2013 Metallo-β-lactamase: inhibitors and reporter substrates. Biochim Biophys Acta 1834:1648–1659. doi:10.1016/j.bbapap.2013.04.024. 23632317

[11] Buynak JD 2013 β-Lactamase inhibitors: a review of the patent literature (2010–2013). Expert Opin Ther Pat 23:1469–1481. doi:10.1517/13543776.2013.831071. 23967802

[12] Li N, Xu Y, Xia Q, Bai C, Wang T, Wang L, He D, Xie N, Li L, Wang J, Zhou HG, Xu F, Yang C, Zhang Q, Yin Z, Guo Y, Chen Y 2014 Simplified captopril analogues as NDM-1 inhibitors. Bioorg Med Chem Lett 24:386–389. doi:10.1016/j.bmcl.2013.10.068. 24269122

[13] Day JA, Cohen SM 2013 Investigating the selectivity of metalloenzyme inhibitors. J Med Chem 56:7997–8007. doi:10.1021/jm401053m. 24074025 PMC3880651

[14] Ma J, Cao Q, McLeod SM, Ferguson K, Gao N, Breeze AL, Hu J 2015 Target-based whole-cell screening by ^1^H NMR spectroscopy. Angew Chem Int Ed Engl 54:4764–4767. doi:10.1002/anie.201410701. 25693499 PMC4471574

[15] Nauton L, Kahn R, Garau G, Hernandez JF, Dideberg O 2008 Structural insights into the design of inhibitors for the L1 metallo-β-lactamase from Stenotrophomonas maltophilia. J Mol Biol 375:257–269. doi:10.1016/j.jmb.2007.10.036. 17999929

[16] Badarau A, Page MI 2006 The variation of catalytic efficiency of Bacillus cereus metallo-β-lactamase with different active site metal ions. Biochemistry 45:10654–10666. doi:10.1021/bi060934l. 16939217

[17] Heinz U, Bauer R, Wommer S, Meyer-Klaucke W, Papamichaels C, Bateson J, Adolph HW 2003 Coordination geometries of metal ions in d- or l-captopril-inhibited metallo-β-lactamases. J Biol Chem 278:20659–20666. doi:10.1074/jbc.M212581200. 12668674

[18] Lienard BM, Garau G, Horsfall L, Karsisiotis AI, Damblon C, Lassaux P, Papamicael C, Roberts GC, Galleni M, Dideberg O, Frère JM, Schofield CJ 2008 Structural basis for the broad-spectrum inhibition of metallo-β-lactamases by thiols. Org Biomol Chem 6:2282–2294. doi:10.1039/b802311e. 18563261

[19] Rydzik AM, Brem J, van Berkel SS, Pfeffer I, Makena A, Claridge TD, Schofield CJ 2014 Monitoring conformational changes in the NDM-1 metallo-β-lactamase by ^19^F NMR spectroscopy. Angew Chem Int Ed Engl 53:3129–3133. doi:10.1002/anie.201310866. 24615874 PMC4499255

[20] Antony J, Gresh N, Olsen L, Hemmingsen L, Schofield CJ, Bauer R 2002 Binding of d- and l-captopril inhibitors to metallo-β-lactamase studied by polarizable molecular mechanics and quantum mechanics. J Comput Chem 23:1281–1296. doi:10.1002/jcc.10111. 12210153

[21] King DT, Worrall LJ, Gruninger R, Strynadka NC 2012 New Delhi metallo-β-lactamase: structural insights into β-lactam recognition and inhibition. J Am Chem Soc 134:11362–11365. doi:10.1021/ja303579d. 22713171

[22] Garcia-Saez I, Hopkins J, Papamicael C, Franceschini N, Amicosante G, Rossolini GM, Galleni M, Frère JM, Dideberg O 2003 The 1.5-A structure of Chryseobacterium meningosepticum zinc β-lactamase in complex with the inhibitor, d-captopril. J Biol Chem 278:23868–23873. doi:10.1074/jbc.M301062200. 12684522

[23] Garcia-Saez I, Mercuri PS, Papamicael C, Kahn R, Frère JM, Galleni M, Rossolini GM, Dideberg O 2003 Three-dimensional structure of FEZ-1, a monomeric subclass B3 metallo-β-lactamase from Fluoribacter gormanii, in native form and in complex with d-captopril. J Mol Biol 325:651–660. doi:10.1016/S0022-2836(02)01271-8. 12507470

[24] Wang YT, Lu CY, Hour TC, Cheng TL 2014 Inhibitor and substrate binding by New Delhi metallo-β-lactamase-1: a molecular dynamics studies. Curr Comput Aided Drug Des 10:197–204. doi:10.2174/1574886309666141126145225#sthash.fxKtMHtA.dpuf. 25479381

[25] Menard PR, Suh JT, Jones H, Loev B, Neiss ES, Wilde J, Schwab A, Mann WS 1985 Angiotensin converting enzyme inhibitors. (Mercaptoaroyl)amino acids. J Med Chem 28:328–332. doi:10.1021/jm00381a012. 2983075

[26] Skiles JW, Suh JT, Williams BE, Menard PR, Barton JN, Loev B, Jones H, Neiss ES, Schwab A 1986 Angiotensin-converting enzyme inhibitors: new orally active 1,4-thiazepine-2,5-diones, 1,4-thiazine-2,5-diones, and 1,4-benzothiazepine-2,5-diones possessing antihypertensive activity. J Med Chem 29:784–796. doi:10.1021/jm00155a032. 3009814

[27] van Berkel SS, Brem J, Rydzik AM, Salimraj R, Cain R, Verma A, Owens RJ, Fishwick CWG, Spencer J, Schofield CJ 2013 Assay platform for clinically relevant metallo-β-lactamases. J Med Chem 56:6945–6953. doi:10.1021/jm400769b. 23898798 PMC3910272

[28] Lassaux P, Traore DA, Loisel E, Favier A, Docquier JD, Sohier JS, Laurent C, Bebrone C, Frere JM, Ferrer JL, Galleni M 2011 Biochemical and structural characterization of the subclass B1 metallo-β-lactamase VIM-4. Antimicrob Agents Chemother 55:1248–1255. doi:10.1128/AAC.01486-09. 21149620 PMC3067066

[29] Otwinowski Z, Minor W 1997 Processing of X-ray diffraction data collected in oscillation mode. Methods Enzymol 276:307–326. doi:10.1016/S0076-6879(97)76066-X. 27754618

[30] McCoy AJ, Grosse-Kunstleve RW, Adams PD, Winn MD, Storoni LC, Read RJ 2007 Phaser crystallographic software. J Appl Crystallogr 40:658–674. doi:10.1107/S0021889807021206. 19461840 PMC2483472

[31] Adams PD, Afonine PV, Bunkoczi G, Chen VB, Davis IW, Echols N, Headd JJ, Hung LW, Kapral GJ, Grosse-Kunstleve RW, McCoy AJ, Moriarty NW, Oeffner R, Read RJ, Richardson DC, Richardson JS, Terwilliger TC, Zwart PH 2010 PHENIX: a comprehensive Python-based system for macromolecular structure solution. Acta Crystallogr D Biol Crystallogr 66:213–221. doi:10.1107/S0907444909052925. 20124702 PMC2815670

[32] Emsley P, Lohkamp B, Scott WG, Cowtan K 2010 Features and development of Coot. Acta Crystallogr D Biol Crystallogr 66:486–501. doi:10.1107/S0907444910007493. 20383002 PMC2852313

[33] Sengerová B, Allerston CK, Abu M, Lee SY, Hartley J, Kiakos K, Schofield CJ, Hartley JA, Gileadi O, McHugh PJ 2012 Characterization of the human SNM1A and SNM1B/Apollo DNA repair exonucleases. J Biol Chem 287:26254–26267. doi:10.1074/jbc.M112.367243. 22692201 PMC3406710

[34] Yong D, Toleman MA, Giske CG, Cho HS, Sundman K, Lee K, Walsh TR 2009 Characterization of a new metallo-β-lactamase gene, bla(NDM-1), and a novel erythromycin esterase gene carried on a unique genetic structure in Klebsiella pneumoniae sequence type 14 from India. Antimicrob Agents Chemother 53:5046–5054. doi:10.1128/AAC.00774-09. 19770275 PMC2786356

[35] El Salabi A, Borra PS, Toleman MA, Samuelsen O, Walsh TR 2012 Genetic and biochemical characterization of a novel metallo-β-lactamase, TMB-1, from an Achromobacter xylosoxidans strain isolated in Tripoli, Libya. Antimicrob Agents Chemother 56:2241–2245. doi:10.1128/AAC.05640-11. 22290947 PMC3346670

[36] Guo Y, Wang J, Niu G, Shui W, Sun Y, Zhou H, Zhang Y, Yang C, Lou Z, Rao Z 2011 A structural view of the antibiotic degradation enzyme NDM-1 from a superbug. Protein Cell 2:384–394. doi:10.1007/s13238-011-1055-9. 21637961 PMC4875342

[37] Vella P, Hussein WM, Leung EWW, Clayton D, Ollis DL, Mitić N, Schenk G, McGeary RP 2011 The identification of new metallo-β-lactamase inhibitor leads from fragment-based screening. Bioorg Med Chem Lett 21:3282–3285. doi:10.1016/j.bmcl.2011.04.027. 21536436

[38] Davey MS, Tyrrell JM, Howe RA, Walsh TR, Moser B, Toleman MA, Eberl M 2011 A promising target for treatment of multidrug-resistant bacterial infections. Antimicrob Agents Chemother 55:3635–3636. doi:10.1128/AAC.00382-11. 21537011 PMC3122435

[39] Concha NO, Janson CA, Rowling P, Pearson S, Cheever CA, Clarke BP, Lewis C, Galleni M, Frère JM, Payne DJ, Bateson JH, Abdel-Meguid SS 2000 Crystal structure of the IMP-1 metallo β-lactamase from Pseudomonas aeruginosa and its complex with a mercaptocarboxylate inhibitor: binding determinants of a potent, broad-spectrum inhibitor. Biochemistry 39:4288–4298. doi:10.1021/bi992569m. 10757977

[40] Davies AM, Rasia RM, Vila AJ, Sutton BJ, Fabiane SM 2005 Effect of pH on the active site of an Arg121Cys mutant of the metallo-β-lactamase from Bacillus cereus: implications for the enzyme mechanism. Biochemistry 44:4841–4849. doi:10.1021/bi047709t. 15779910

[41] Galleni M, Lamotte-Brasseur J, Rossolini GM, Spencer J, Dideberg O, Frere JM 2001 Standard numbering scheme for class B β-lactamases. Antimicrob Agents Chemother 45:660–663. doi:10.1128/AAC.45.3.660-663.2001. 11181339 PMC90352

[42] Brem J, Struwe WB, Rydzik AM, Tarhonskaya H, Pfeffer I, Flashman E, van Berkel SS, Spencer J, Claridge TD, McDonough MA, Benesch JL, Schofield CJ 2015 Studying the active-site loop movement of the Sao Paolo metallo-β-lactamase-1. Chem Sci 6:956–963. doi:10.1039/C4SC01752H. 25717359 PMC4333608

[43] Garau G, Garcia-Saez I, Bebrone C, Anne C, Mercuri P, Galleni M, Frere JM, Dideberg O 2004 Update of the standard numbering scheme for class B β-lactamases. Antimicrob Agents Chemother 48:2347–2349. doi:10.1128/AAC.48.7.2347-2349.2004. 15215079 PMC434215

[44] Garcia-Saez I, Docquier JD, Rossolini GM, Dideberg O 2008 The three-dimensional structure of VIM-2, a Zn-β-lactamase from Pseudomonas aeruginosa in its reduced and oxidised form. J Mol Biol 375:604–611. doi:10.1016/j.jmb.2007.11.012. 18061205

[45] Zhang H, Hao Q 2011 Crystal structure of NDM-1 reveals a common β-lactam hydrolysis mechanism. FASEB J 25:2574–2582. doi:10.1096/fj.11-184036. 21507902

[46] Merino M, Perez-Llarena FJ, Kerff F, Poza M, Mallo S, Rumbo-Feal S, Beceiro A, Juan C, Oliver A, Bou G 2010 Role of changes in the L3 loop of the active site in the evolution of enzymatic activity of VIM-type metallo-β-lactamases. J Antimicrob Chemother 65:1950–1954. doi:10.1093/jac/dkq259. 20624761

[47] Mojica MF, Mahler SG, Bethel CR, Taracila MA, Kosmopoulou M, Papp-Wallace KM, Llarrull LI, Wilson BM, Marshall SH, Wallace CJ, Villegas MV, Harris ME, Vila AJ, Spencer J, Bonomo RA 2015 Exploring the role of residue 228 in substrate and inhibitor recognition by VIM metallo-β-lactamases. Biochemistry 54:3183–3196. doi:10.1021/acs.biochem.5b00106. 25915520 PMC4700511

[48] Cadag E, Vitalis E, Lennox KP, Zhou CL, Zemla AT 2012 Computational analysis of pathogen-borne metallo β-lactamases reveals discriminating structural features between B1 types. BMC Res Notes 5:96. doi:10.1186/1756-0500-5-96. 22333139 PMC3293060

[49] Edelstein MV, Skleenova EN, Shevchenko OV, D'Souza JW, Tapalski DV, Azizov IS, Sukhorukova MV, Pavlukov RA, Kozlov RS, Toleman MA, Walsh TR 2013 Spread of extensively resistant VIM-2-positive ST235 Pseudomonas aeruginosa in Belarus, Kazakhstan, and Russia: a longitudinal epidemiological and clinical study. Lancet Infect Dis 13:867–876. doi:10.1016/S1473-3099(13)70168-3. 23845533

[50] Meini MR, Llarrull LI, Vila AJ 20 8 2015 Overcoming differences: the catalytic mechanism of metallo-β-lactamases. FEBS Lett doi:10.1016/j.febslet.2015.08.015. PMC4640939 26297824

