# Supplementary material for: Structural Basis of Metallo-β-Lactamase Inhibition by Captopril Stereoisomers
Source: Antimicrob Agents Chemother. 2015 Dec 31;60(1):142–50. doi: 10.1128/AAC.01335-15 (PMC4704194; doi:10.1128/AAC.01335-15)
Supplement: Supplemental material [file supp_60_1_142__index.html]

Structural Basis of Metallo-β-Lactamase Inhibition by Captopril Stereoisomers — Supplemental material 

# Structural Basis of Metallo-β-Lactamase Inhibition by Captopril Stereoisomers

## Supplemental material

- Supplemental file 1 -

  Supplemental figures, schemes, and tables.

  PDF, 2.1M
