# Supplementary material for: Structural Basis of Metallo-β-Lactamase Inhibition by Captopril Stereoisomers
Source: Antimicrob Agents Chemother. 2015 Dec 31;60(1):142–50. doi: 10.1128/AAC.01335-15 (PMC4704194; doi:10.1128/AAC.01335-15)
Supplement: Supplemental material [file AAC.01335-15_zac001164695so1.pdf]

Supplemental material

Structural basis of metallo-β-lactamase inhibition by captopril stereoisomers

Jürgen Brem[1*], Sander S. van Berkel[1*], David Zollman[1*], Sook Y. Lee[1], Opher Gileadi[2], Peter J. McHugh[3], Timothy R. Walsh[4], Michael A. McDonough[1#], and Christopher J. Schofield[1#]

[1] Department of Chemistry, University of Oxford, 12 Mansfield Road, Oxford, OX1 3TA, United Kingdom.

[2] Structural Genomics Consortium, University of Oxford, Old Road Campus, Headington, Oxford OX3 7BN, United Kingdom.

[3] Department of Oncology, Weatherall Institute of Molecular Medicine, University of Oxford, John Radcliffe Hospital, Oxford OX3 9DS, United Kingdom.

[4] Department of Microbiology and Infectious Diseases, Institute of Infection and Immunity, Heath Hospital, Cardiff, CF14 4XN, United Kingdom.

* Co-first authors: Jürgen Brem, Sander S. van Berkel and David Zollman

[#] Corresponding Authors: Michael A. McDonough, Department of Chemistry, University of Oxford, 12 Mansfield Road, Oxford, OX1 3TA, United Kingdom, michael.mcdonough@chem.ox.ac.uk and Christopher J. Schofield, Department of Chemistry, University of Oxford, 12 Mansfield Road, Oxford, OX1 3TA, United Kingdom, christopher.schofield@chem.ox.ac.uk

Content:

A) List of Figures and Schemes page S3

B) List of Tables page S14

C) Experimental section for synthesis page S19

References page S25

A) List of Figures and Schemes

Supplemental material Figure S1. Some reported MBL inhibitors.

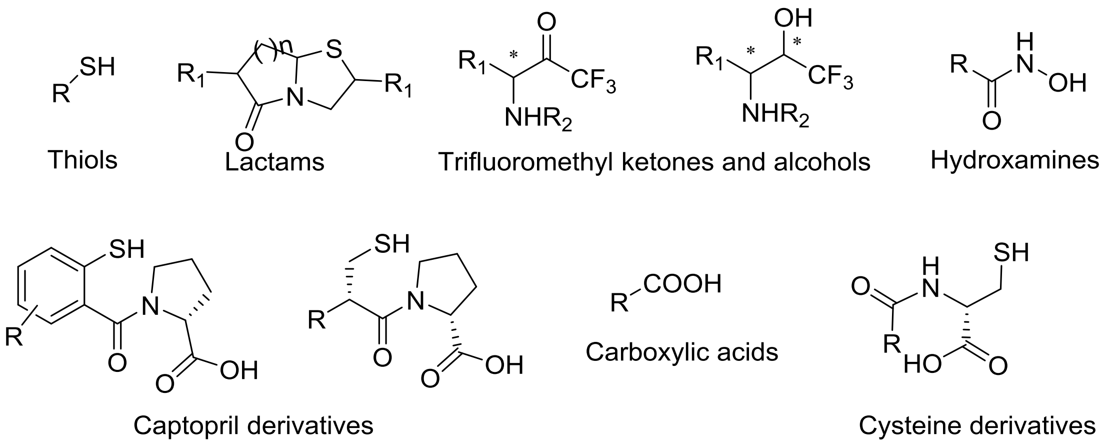

Supplemental material Figure S2. Views from reported crystal structures of MBLs with L- or D-captopril. Overlay of previously reported structures: NDM-1(B1) with L-captopril (PDB: 4EXS), BlaB(B1) with D-captopril (PDB: 1M2X), CphA(B2) with D-captopril (PDB: 2QDS), L-1(B3) with D-captopril (PDB: 2FU8), FEZ-1(B3) with D-captopril (PDB: 1JT1). The MBL backbone is in grey, the residues involved in the binding of Zn(II) in the active site are in light blue. Zn(II) ions are represented by dark gray spheres. The absolute configurations of D- and L-captopril are depicted for each structure. The different captopril stereoisomers are displayed in sticks and in yellow or orange.

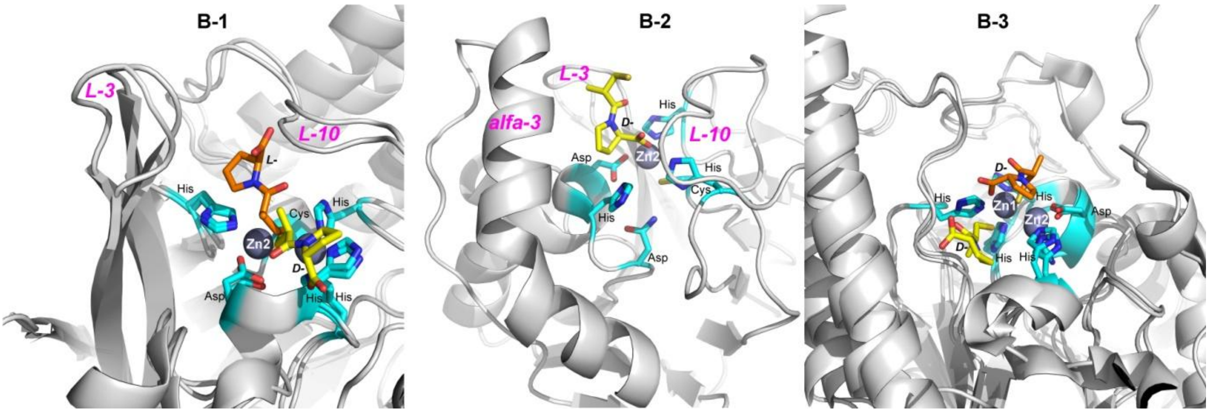

Supplemental material Figure S3. Overlay of the MBL crystal structures of L- or D-captopril. Overlay of views from crystal structures of NDM-1, BcII, IMP-1 and VIM-2 complexed with L-captopril. B) Overlay of a view from the crystal structure of NDM-1 complexed with L-captopril and crystal structures of BlaB, BcII, IMP-1 and VIM-2 complexed with D-captopril (notice the opposite orientation in the case of the D-captopril BlaB structure). Zinc ions are represented by dark grey spheres; D- and L-captopril ligands are in red and yellow, respectively. The MBL backbone is in grey and the flexible active site loops are in pink (Loops L-3 and L-10).

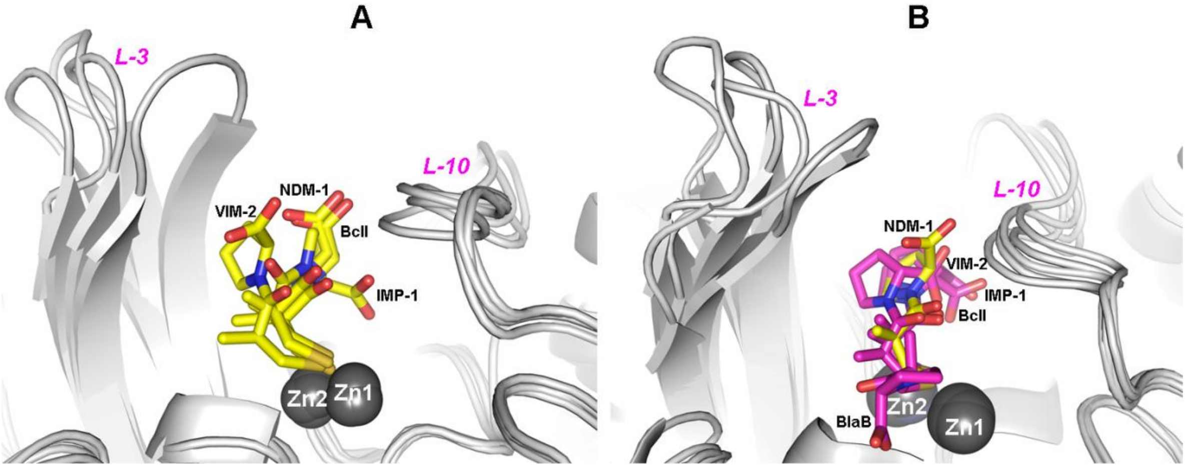

Supplemental material Figure S4. Protein-ligand interactions between various MBLs and captopril (different stereoisomer) described using LIGPLOT (1). Hydrogen bonding interactions are shown as lines and ligand-protein hydrophobic contacts are as curved combs.

BlaB - D-Captopril

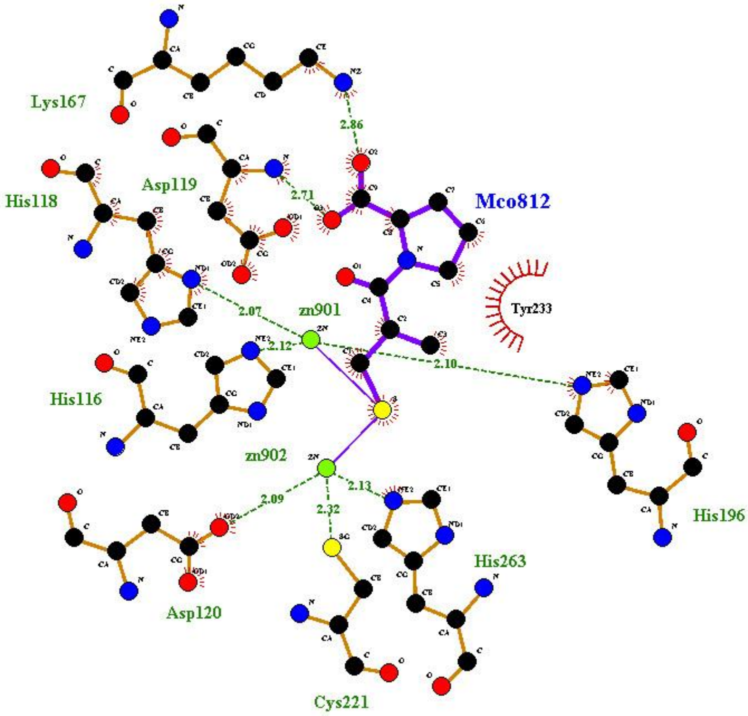

NDM-1 - L-Captopril

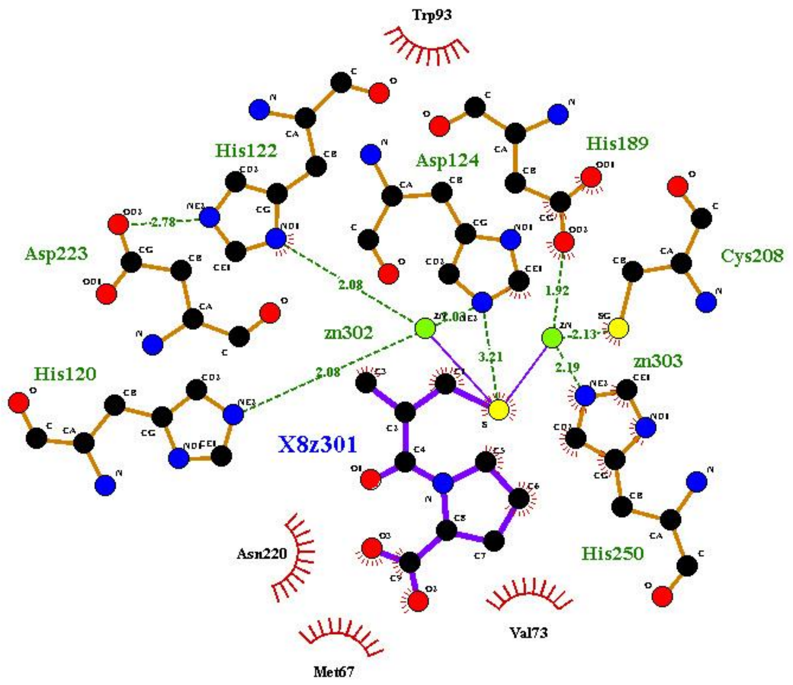

CphA - D-Captopril

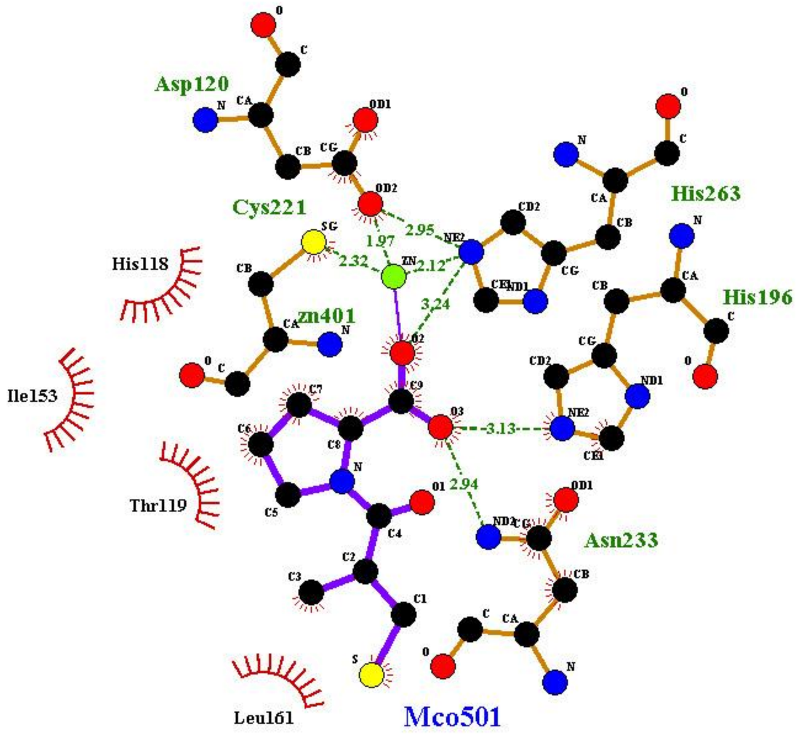

Fez-1 - D-Captopril

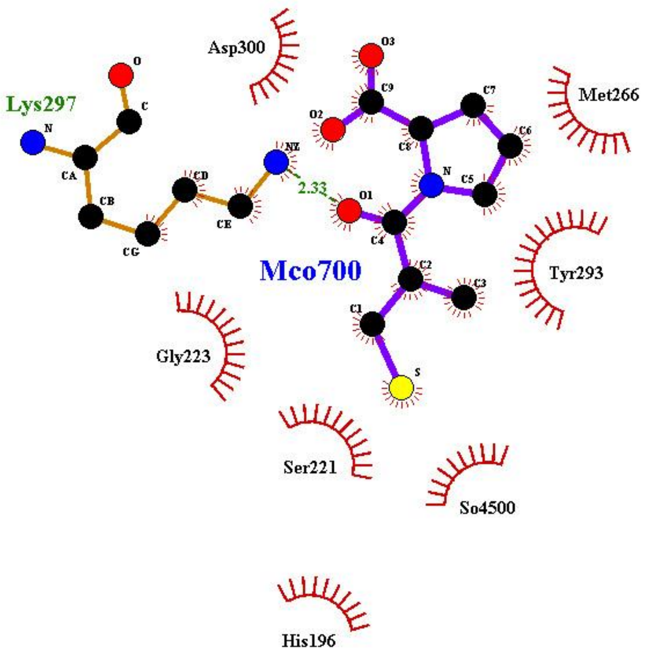

L1 - D-Captopril

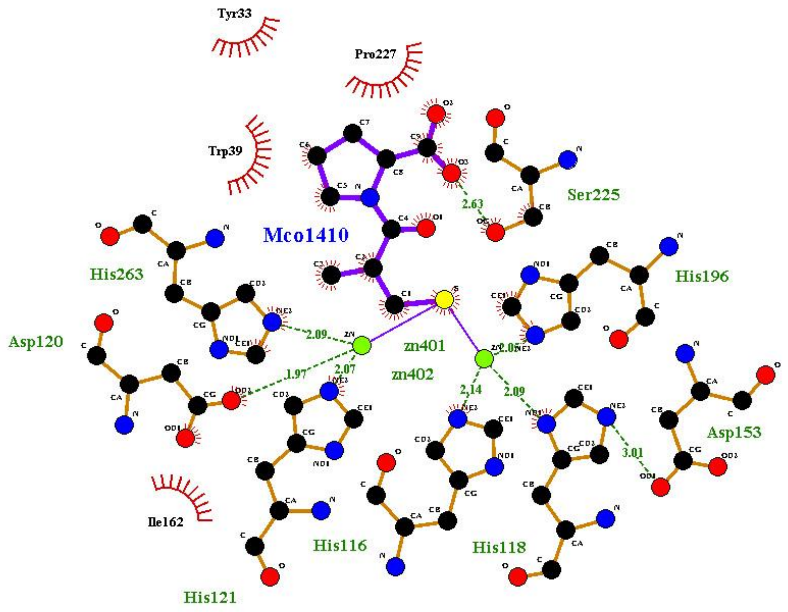

BcII - L-captopril

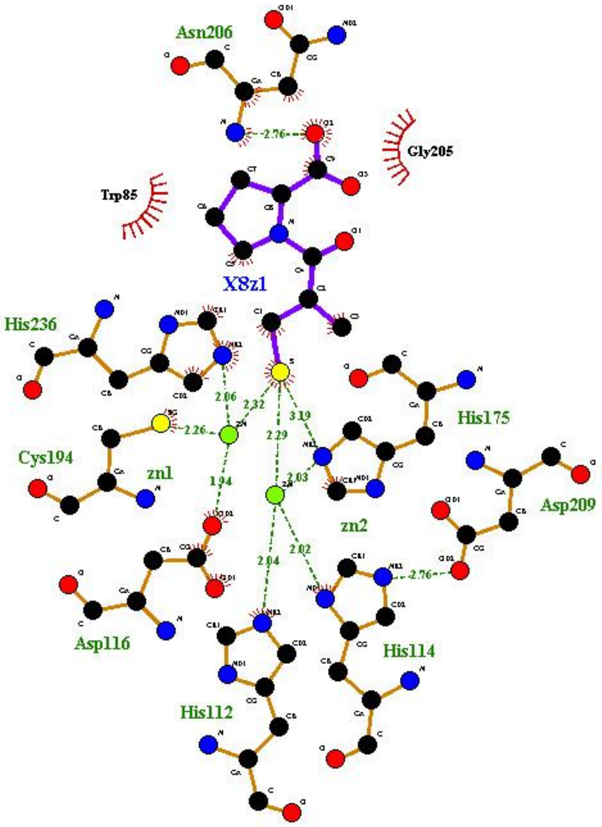

BcII - D-captopril

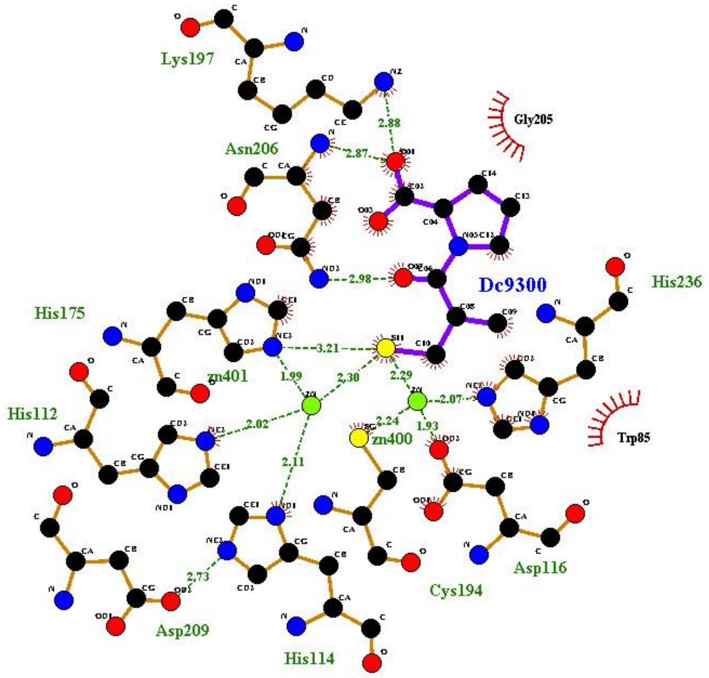

IMP-1 - L-Captopril

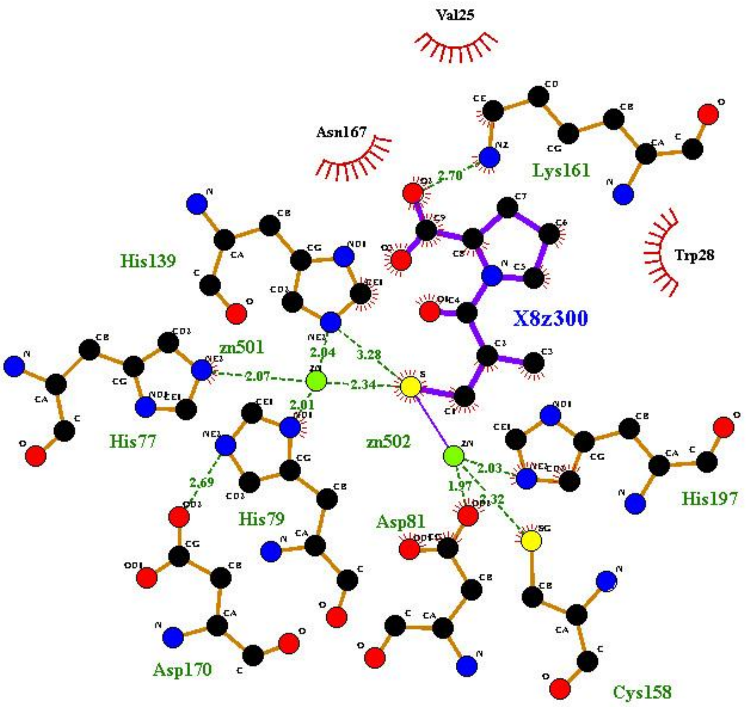

IMP-1 - D-Captopril

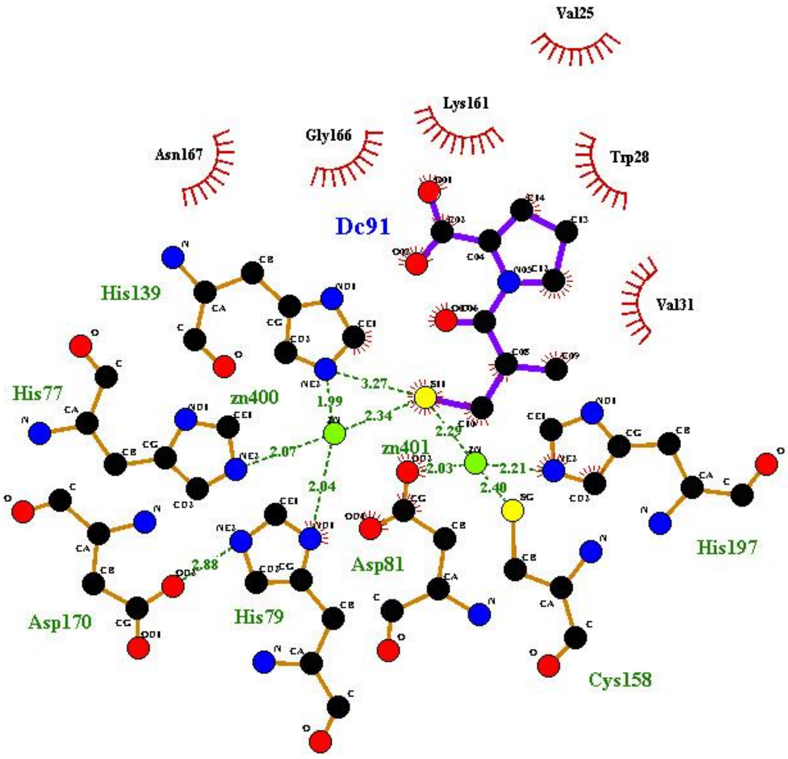

Vim-2 -   L-Captopril

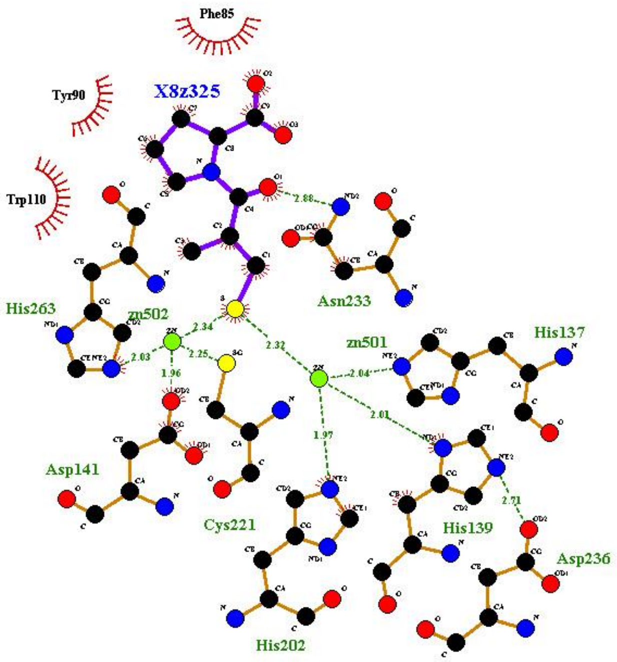

Vim-2 -   D-Captopril

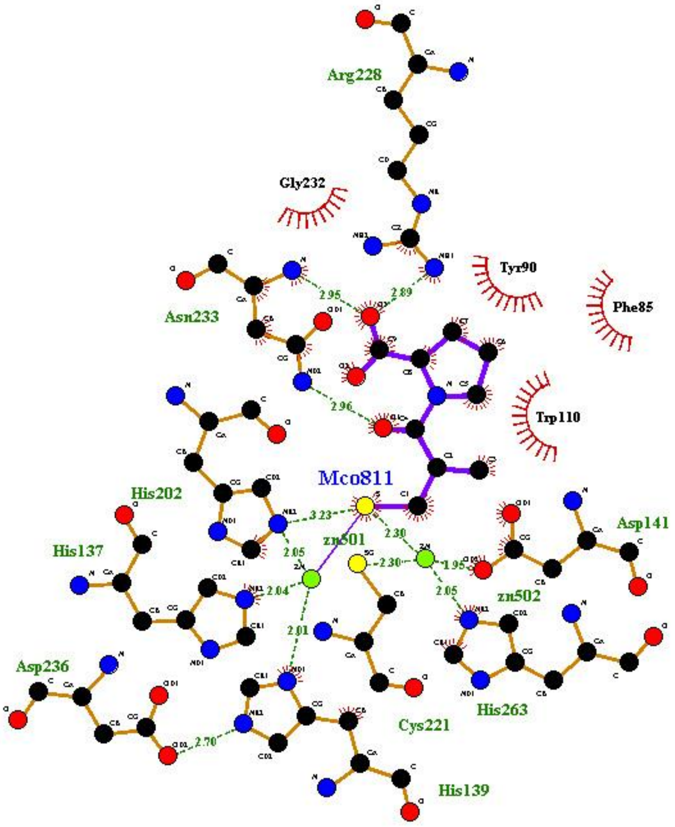

Supplemental material Figure S5. Example of assay results used to test for DCLRE1A and DCLRE1B nuclease inhibition by D-captopril; for details of assay conditions see Material and methods section. [SNM1A] = [SNM1B] = 0.8 nM; [21nt fluor-ssDNA] = 1 µM, assay time = 20 mins at 37 °C, 20% denaturing gel; Captopril = D-captopril.

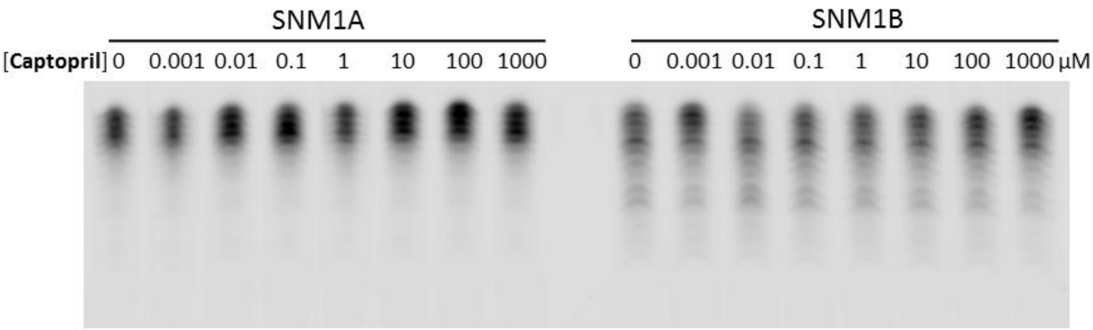

Supplemental material Figure S6. View from a VIM-2 D-captopril complex structure overlaid with (a) $2F_o$-F_c density contoured at 1 σ (blue mesh) and F_o-F_c density contoured at 3 σ (green mesh). Density was calculated from the final model, following the omission of the ligand, against experimental diffraction data.

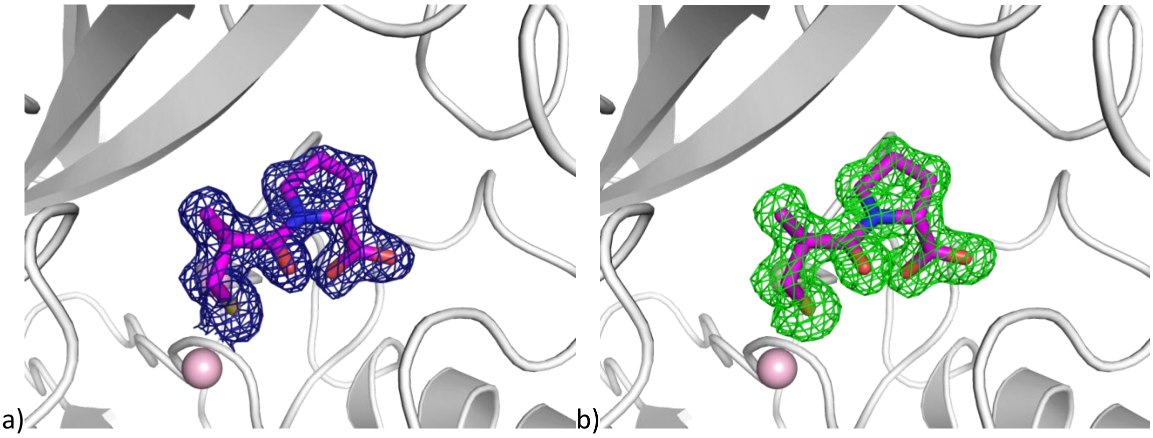

Supplemental material Figure S7. Crystallographically observed water displacement upon inhibitor binding to BcII, IMP-1 and VIM-2. Water molecules are shown as green (apo-di-Zn(II)-MBLs) blue (L-captopril bound to MBLs) and red (D-captopril bound to MBLs) spheres. Black arrows show the water molecules involved in hydrogen bonding.

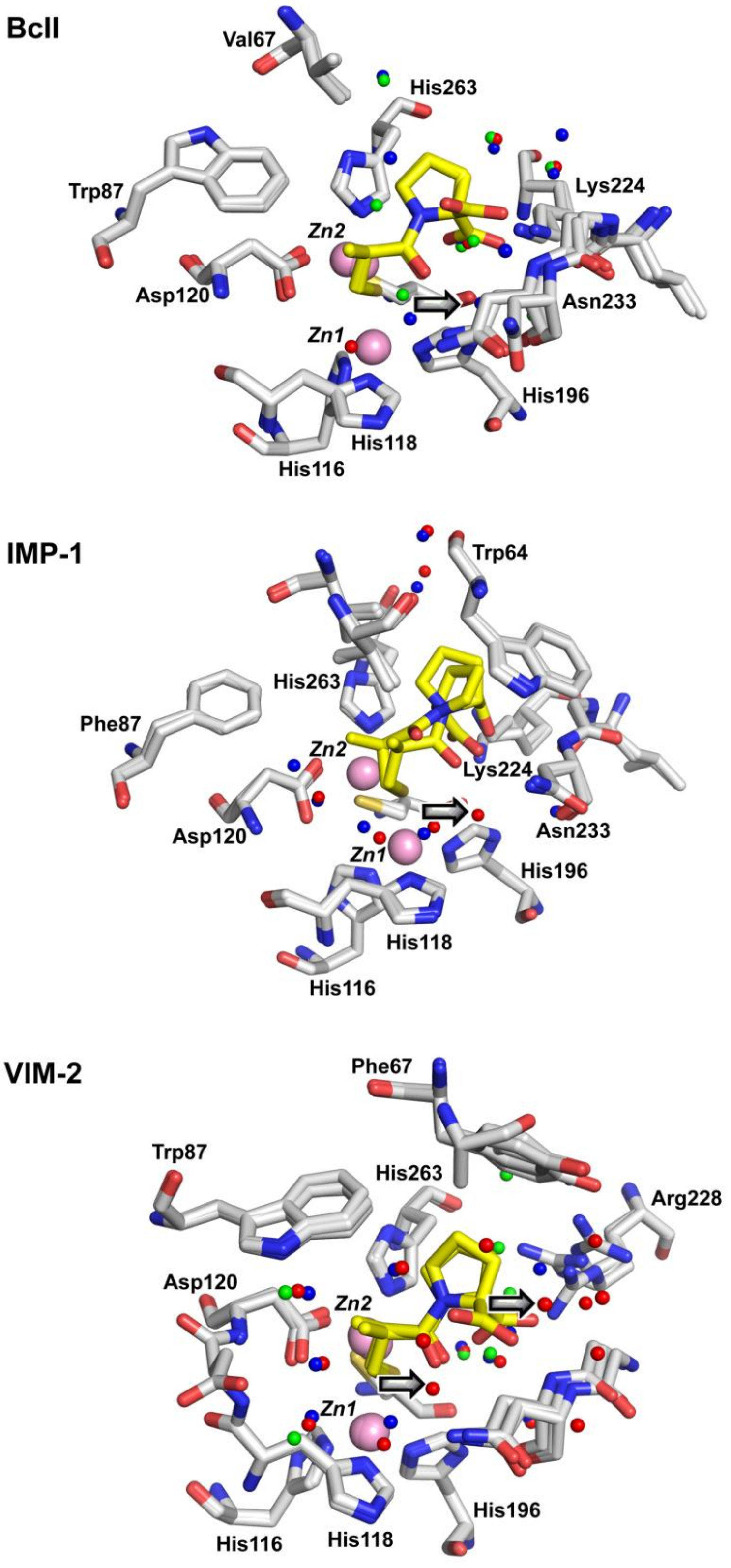

Synthesis of captopril stereoisomers

The syntheses of D-captopril (**2**), *epi*-L-captopril (**3**), and *epi*-D-captopril (**4**) were synthesised using reported procedures (2-4) as shown in Scheme S1. L-Captopril (**1**) is commercially available (Sigma-Aldrich).

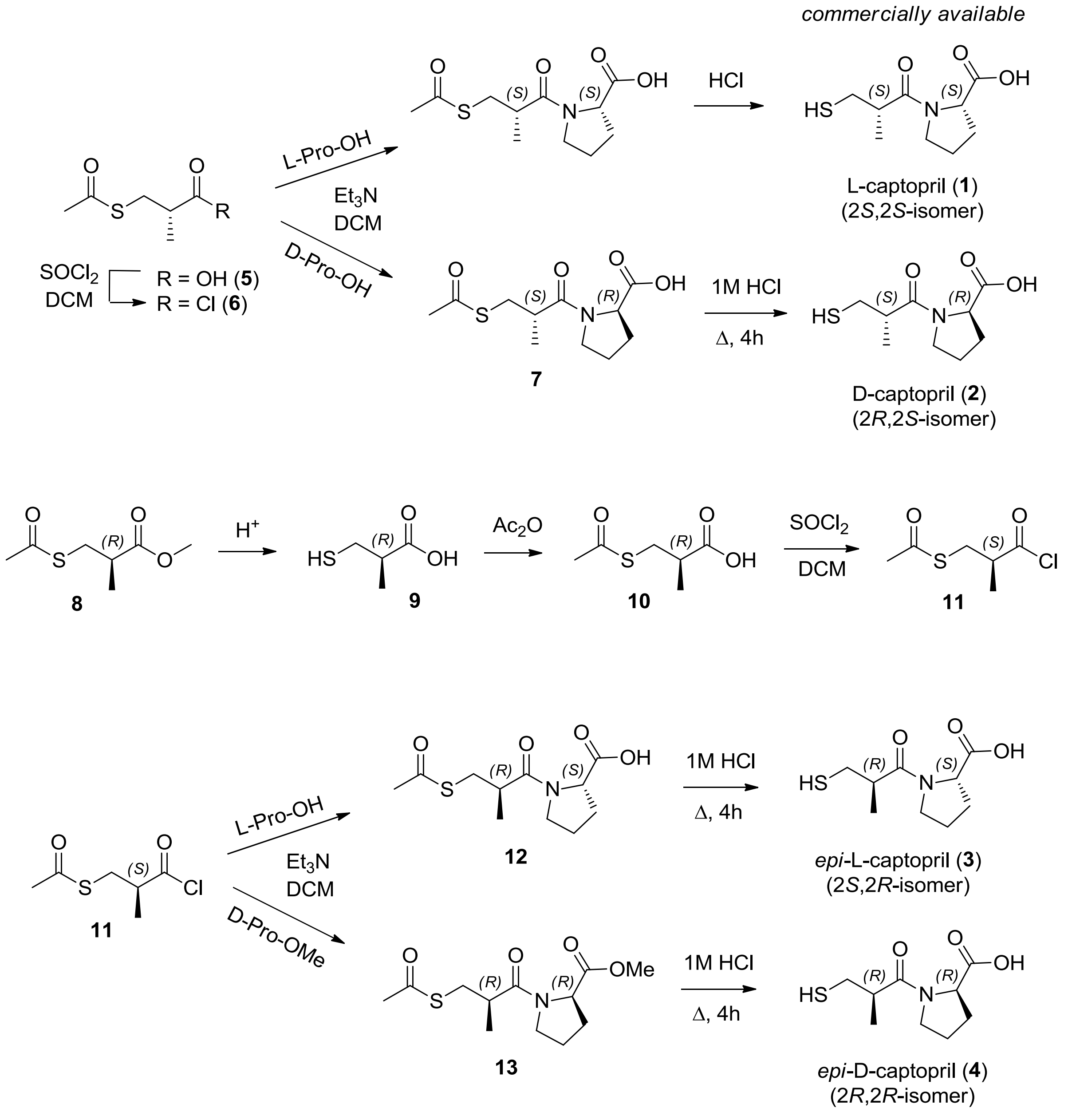

Supplemental material Scheme S1. Synthesis of D-captopril (**2**), *epi*-L-captopril (**3**) and *epi*-D-captopril (**4**).

The mercaptobenzoyl proline (MBP) derivatives (**17** and **19**) was carried out using reported procedures (5) as show in Scheme S2.

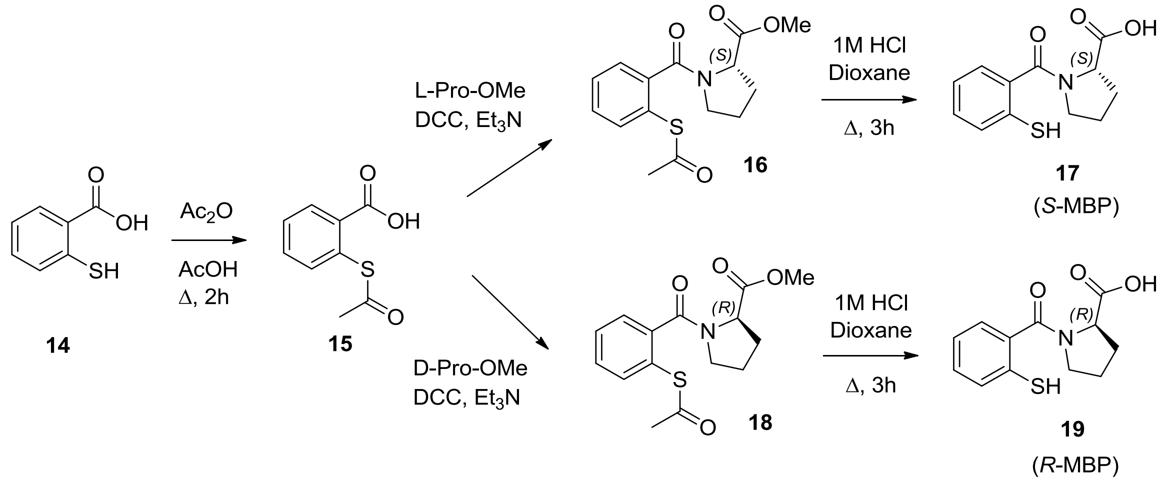

Supplemental material Scheme S2. Synthesis of captopril derivatives *S*-MBP (**17**) and *R*-MBP (**19**).

B) List of Tables

Supplemental material Table S1. Maximum percentage sequence identities for MBLs used in this study.

	**BcII**	**IMP-1**	**SPM-1**	**VIM-2**	**NDM-1**
**BcII**	-	38	28	38	33
**IMP-1**	38	-	34	33	34
**SPM-1**	27	34	-	30	26
**VIM-2**	38	33	30	-	37
**NDM-1**	33	34	27	37	-

Supplemental material Table S2. Crystallization conditions.

	**Sample composition**	**Crystallization condition**	**Vapour diffusion conditions**
apo-di-Zn(II) BcII	BcII in crystallization buffer + 1 mM TCEP	0.1 M Bis-Tris pH = 5.5, 19 % PEG 3350, 0.05 M ammonium sulfate	Sitting drop, protein-to-well ratio, 1:2, 293K
D-captopril BcII	BcII in crystallization buffer + TCEP (1 mM) + D-captopril (7.5 mM)	0.1 M Bis-Tris pH = 5.5, 23 % PEG 3350, 0.1 M ammonium sulfate	Sitting drop, protein-to-well ratio, 1:2, 293K
L-captopril BcII	BcII in crystallization buffer + TCEP (1 mM) L-captopril (7.5 mM)	0.1 M Bis-Tris pH = 5.5, 17 % PEG 3350, 0.15 M ammonium sulfate	Sitting drop, protein-to-well ratio, 1:2, 293K
D-captopril IMP-1	IMP-1 in crystallization buffer + DTT (1 mM) + D-captopril (2.5 mM)	0.1 M sodium acetate pH 4.5, 23% PEG 8000, 0.05 M $LiSO_4$	Sitting drop, protein-to-well ratio, 1:1, 293K
L-captopril IMP-1	IMP-1 in crystallization buffer L-captopril (2.5 mM)	0.1 M sodium acetate pH 4.5, 23% PEG 8000, 0.2 M $LiSO_4$	Sitting drop, protein-to-well ratio, 1:2, 293K
apo-di-Zn(II) VIM-2	VIM-2 in crystallization buffer + 1 mM TCEP	0.25 M magnesium formate, 22% % PEG 3350	Sitting drop, protein-to-well ratio, 1:1, 293K
D-captopril VIM-2	VIM-2 in crystallization buffer + TCEP (1 mM) + D-captopril (4.5 mM)	0.1 magnesium formate, 25% PEG 3350	Sitting drop, protein-to-well ratio, 1:2, 293K
L-captopril VIM-2	VIM-2 in crystallization buffer + TCEP (1 mM) L-captopril (5.0 mM)	0.1 magnesium formate, 23% PEG 3350	Sitting drop, protein-to-well ratio, 1:2, 293K

Supplemental material Table S3. Data Collection and Refinement Statistics for VIM-2

Dataset	VIM-2 di-Zn(II)	VIM-2 + D-captopril	VIM-2 + L-captopril
**Processing**			
X-ray source	I04-1	FRE+	I03
Space group	$C2_1$	$C2_1$	$C2_1$
Unit cell dimensions a, b, c (Å)	103.0, 79.7, 68.1	103.9, 79.0, 67.2	102.2, 79.4, 67.8
Unit cell dimensions α, β, γ (deg)	90, 130.6, 90	90.0, 131.6, 90.0	90.0, 130.3, 90.0
Mol/ASU†	2	2	2
Resolution (outer shell) (Å)	50.0-1.30 (1.35-1.30)	50.0-1.40 (1.45-1.40)	50.0-1.20 (1.24-1.20)
Completeness (outer shell) (%)	98.2 (97.0)	98.7 (95.3)	99.1 (91.0)
R_{merge} (outer shell)	11.0 (25.3)	10.0 (34.7)	17.1 (61.2)
No. of unique reflections	101604	78943	126706
I/σ mean (outer shell)	10.9 (3.5)	10.7 (2.0)	10.6 (1.9)
Wilson B	9.93	13.46	13.24
**Refinement**			
B factors			
Overall	15.34	17.02	18.66
Protein (Chain A, B)	13.45, 13.85	15.29, 15.51	17.24, 17.53
Ligand (Chain A, B)	~	32.03, 32.03	18.31, 21.07
Water (Chain A, B)	28.29, 27.63	27.33, 27.53	28.74, 29.91
RMSD from ideal bond length (Å) ‡	0.025	0.024	0.025
RMSD from ideal angles (deg)	1.239	1.420	1.403
$R_{work\ (\%)}$	11.3	13.2	14.3
$R_{free\ (\%)}$	13.8	16.7	17.2

I04-1 and I03 are beamlines at Diamond Light Source Oxford, FRE+ is an in house CCD detector. †Mol/ASU = molecules per asymmetric unit. ‡ RMSD = root mean square deviation.

Supplemental material Table S4. Data Collection and Refinement Statistics for IMP-1

Dataset	IMP-1 D-captopril	IMP-1 L-captopril
**Processing**		
X-ray source	I03	I03
Space group	$P2_12_12_1$	$P2_12_12_1$
Unit cell dimensions a, b, c (Å)	194.6, 50.2, 54.6	193.6, 49.9, 54.6
Unit cell dimensions α, β, γ (deg)	90.0, 90.0, 90.0	90.0, 90.0, 90.0
Mol/ASU	2	2
Resolution (outer shell) (Å)	64.86-1.71 (1.76-1.71)	52.5-2.01 (2.08-2.01)
Completeness (outer shell) (%)	100 (100)	99.9 (99.1)
R_{merge} (outer shell)	8.6 (63.1)	9.3 (35.3)
No. of unique reflections	58579	36158
I/σ mean (outer shell)	13.7 (3.2)	9.7 (3.8)
Wilson B	22.06	27.43
**Refinement**		
B factor		
Overall	47.64	44.53
Protein (Chain A, B)	25.06, 77.04	21.79, 69.76
Ligand (Chain A only)	32.03	37.47
Water (Chain A, B)	39.23, 51.46	34.43, 47.38
RMSD from ideal bond length (Å) ‡	0.020	0.004
RMSD from ideal angles (deg)	1.730	0.766
$R_{work\ (\%)}$	14.5	18.4
$R_{free\ (\%)}$	17.6	22.4

I03 is a beamline at Diamond Light Source Oxford. †Mol/ASU = molecules per asymmetric unit. ‡ RMSD = root mean square deviation.

Supplemental material Table S5. Data Collection and Refinement Statistics for BcII

Dataset	BcII di-Zn(II)	BcII D-captopril	BcII L-captopril
**Processing**			
X-ray source	I03	I03	I03
Space group	$C2_1$	$C2_1$	$C2_1$
Unit cell dimensions a, b, c (Å)	53.3, 62.0, 69.5	53.1, 61.5, 69.5	53.0, 61.3, 69.6
Unit cell dimensions α, β, γ (deg)	90.0, 93.0, 90.0	90.0, 93.0, 90.0	90.0, 93.0, 90.0
Mol/ASU	1	1	1
Resolution (outer shell) (Å)	34.7-1.20 (1.23-1.20)	40.2-1.18 (1.21-1.18)	50.0-1.10 (1.14-1.10)
Completeness (outer shell) (%)	98.3 (95.6)	95.0 (90.4)	99.0 (92.5)
R_{merge} (outer shell)	5.7 (51.1)	5.1 (59.5)	8.7 (18.7)
No. of unique reflections	69108	69108	89049
I/σ mean (outer shell)	9.1 (2.0)	11.5 (2.3)	18.1 (5.0)
Wilson B	14.14	13.60	12.06
**Refinement**			
B factor			
Overall	20.23	20.41	18.16
Protein	18.49	18.63	16.63
Ligand	~	32.03	17.44
Water	31.00	31.23	27.71
RMSD from ideal bond length (Å) [‡]	0.015	0.010	0.009
RMSD from ideal angles (deg)	1.520	1.430	1.360
$R_{work (\%)}$	11.9	12.0	13.2
$R_{free (\%)}$	13.9	13.6	13.9

I03 is a beamline at Diamond Light Source Oxford. [†]Mol/ASU = molecules per asymmetric unit. [‡] RMSD = root mean square deviation.

C) Experimental section for synthesis

Material and Methods for synthesis

Except where stated, chemicals were from commonly used suppliers (Aldrich, Acros, Alfa Aesar, and TCI) and used without purification. Solvents (including dry solvents) for chemical transformations, work-up and chromatography were from Aldrich (Dorset, UK) at HPLC grade, and used without distillation. Silica gel 60 F254 analytical thin layer chromatography (TLC) plates were from Merck (Darmstadt, Germany) and visualized under UV light, or with potassium permanganate ($KMnO_4$) stain. Chromatographic purifications were performed using Merck Geduran 60 silica (40-63 µm) or prepacked SNAP columns on a Biotage SP1 Purification system (Uppsala, Sweden). Deuterated solvents were obtained from Sigma and Apollo Scientific Ltd. ^{1}H and ^{13}C NMR spectra were recorded using a Bruker Avance 400 MHz spectrometer. Chemical shifts are in ppm relative to the solvent peak (6), and coupling constants (*J*) are reported in Hz to the nearest 0.5 Hz. Where conformational isomers were present only the major signals are reported. Low Resolution (LRMS) mass spectrometry data (m/z) were obtained using a Waters LCT Premier instrument with an ESI source and Time of Flight (TOF) analyzer. Fourier transform Infrared (FT-IR) spectra were recorded on a Bruker Tensor 27 instrument. Absorption spectra were recorded on a Varian Cary 4000 UV-Vis spectrophotometer using an 1 mL quartz cuvette. Optical rotations were recorded on a Perkin Elmer 241 Polarimeter. HPLC analysis was run on a Waters Acquity UPLC equipped with a Phenomanex Luna 5 µM C18 column (75 x 4.60 mm) using a gradient of 100% solvent A → 100% solvent B (solvent A: 10% MeCN in H_2O containing 0.05% formic acid; solvent B: 100% MeCN containing 0.1% formic acid), flow rate = 0.6 ml/min and UV detection at 254 nm.

Synthesis

(2*R*)-1-((2*S*)-3-(Acetylthio)-2-methylpropanoyl)pyrrolidine-2-carboxylic acid (**7**) (3)

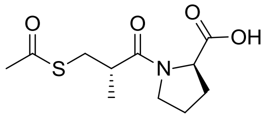

7 was prepared according to the reported procedure (2): (*S*)-3-thio-2-methyl-propionic acid (**5**, 1.62 g, 10 mmol) was dissolved in CH_2Cl_2 (20 mL) and cooled to 0 °C. Thionyl chloride (725 µL, 10 mmol) was added dropwise; the reaction was slowly warmed to room temperature (r.t.), then stirred overnight. Volatiles were removed *in vacuo* and the remaining oil re-dissolved in CH_2Cl_2, which was evaporated to remove any remaining $SOCl_2$. The crude acetyl chloride (6) was dissolved in CH_2Cl_2 (20 mL), afterwhich D-Pro-OH (1.15 g, 10 mmol) was added, followed by the addition of Et_3N (4,18 mL, 30 mmol). The reaction was stirred at r.t. for 16 hours. The reaction was quenched by the addition of 1M HCl (20 mL). After extraction and separation of the layers, the organic layer was dried over Na_2SO_4, filtered and concentrated *in vacuo*. The crude product was purified by column chromatography (CH_2Cl_2/MeOH/AcOH, 9:1:0.05) to give the **7** as a colorless oil (453 mg, 17%). R_F = 0.3 (CH_2Cl_2/MeOH, 9:1:0.05). Analytical data were in accord with literature values (3). ^{1}H NMR (400 MHz, $CDCl_3$) (Signals for major conformational isomer are reported) δ = 4.62 (dd, *J* = 8.0, 2.5 Hz, 1H), 3.75 (ddd, *J* = 10.0, 7.5, 3.0 Hz, 1H), 3.48 (td, *J* = 9.5, 7.0 Hz, 1H), 3.10-3.17 (m, 1H), 2.95-3.01

(m, 1H), 2.89 (dq, J = 14.0, 7.0 Hz, 1H), 2.53 (ddd, J = 12.0, 6.0, 3.0 Hz, 1H), 2.35 (s, 3H), 1.91-2.14 (m, 3H), 1.25 (d, J = 7.0 Hz, 3H) ppm. LRMS calcd. for $C_{11}H_{16}NO_4S$, M-H = 258.08; found; 258.1.

(2*R*)-1-((2*S*)-3-Mercapto-2-methylpropanoyl)pyrrolidine-2-carboxylic acid (D-captopril, **2**) (3)

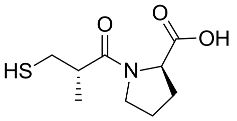

2 was prepared according to the literature procedure (3): (2*R*)-1-((2*S*)-3-(acetylthio)-2-methylpropanoyl)-pyrrolidine-2-carboxylic acid (**7**, 453 mg, 1.75 mmol) was dissolved in 1M HCl (15 mL), then heated to 90 °C for 4 hours. The reaction was cooled to r.t., then extracted with CH_2Cl_2 (3 x 20 mL). The combined organic layers were dried over Na_2SO_4, filtered, and concentrated *in vacuo*. The crude product was purified by column chromatography (CH_2Cl_2/MeOH/AcOH, 9:1:0.05) to give **2** as a colorless oil (220 mg, 58%). R_F = 0.3 (CH_2Cl_2/MeOH, 9:1:0.05) (Clear change in UV activity but no change in R_F). Analytical data were in accord with literature values (3). ^{1}H NMR (400 MHz, $CDCl_3$) (Signals for major conformational isomer are reported) δ = 4.58-4.63 (m, 1H), 3.88-3.83 (m, 1H), 3.57- 3.50 (m, 1H), 2.83-2.99 (m, 2H), 2.38-2.52 (m, 2H), 2.00-2.15 (m, 2H), 1.68 (dd, J = 10.5, 7.5 Hz, 1H), 1.22 (d, J = 6.5 Hz, 3H) ppm. LRMS calcd. for $C_9H_{14}NO_3S$, M-H = 216.07, mass found; 216.1.

(2*R*)-3-Mercapto-2-methylpropanoic acid (**9**) and (2*R*)-3-(Acetylthio)-2-methylpropanoic acid (**10**) (4)

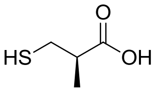

(**9**) and 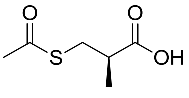

(**10**)

9 was prepared according to the literature procedure (4): (2*R*)-methyl 3-(acetylthio)-2-methylpropanoate (**8**, 880 mg, 5.0 mmol) was dissolved in a mixture of 1M HCl (15 mL) and THF (1 mL) and heated to 95 °C. The reaction was stirred at the indicated temperature for 4 hours (full conversion according to TLC analysis), after which the reaction was cooled to r.t. and extracted with CH_2Cl_2 (3 x 20 mL). The combined organic layers were dried over Na_2SO_4, filtered and concentrated *in vacuo* to yield the desired compound **9** as a colorless oil (595 mg, 99%). R_F = 0.15 (cHex/EtOAc, 1:1). The crude (2*R*)-3-Mercapto-2-methylpropanoic acid was used in the next step without purification.

Acylation of crude (2*R*)-3-mercapto-2-methylpropanoic acid (**9**), was performed according to the literature procedure (4). To a solution of 9 (595 mg, 5.0 mmol) in H_2O (15 mL), cooled to 0 °C, were added in three portions Ac_2O (1.42 g, 15.0 mmol) and 1M NaOH (3 x few drops). The reaction was stirred at until starting material was completely consumed as judged by TCL (4 hours). The reaction was acidified to pH 2 by the addition of HCl (1M) and extracted with CH_2Cl_2 (2 x 30 mL). The combined organic layers were dried over Na_2SO_4, filtered and concentrated *in vacuo*. The crude product was purified by column chromatography (cHex/EtOAc, 1:1→ 1:3) to give (**10**) as colorless oil (692 mg, 85%). R_F = 0.15 (cHex/EtOAc, 1:1). (Note **10** has a similar R_F as compound **9**, but with a different staining behavior with $KMnO_4$). ^{1}H NMR (400 MHz, $CDCl_3$) δ = 12.32-9.43 (app. br. s, 1H), 3.00-3.20 (m, 2H), 2.69-2.79 (m, 1H), 2.35 (s, 3H) ppm. ^{13}C NMR (100 MHz, $CDCl_3$) δ = 195.5,

181.0, 39.9, 31.2, 30.6, 16.7 ppm. LRMS (m/z) calcd. for $C_6H_{10}NaO_3S$, M+Na = 185.20, mass found M+Na = 185.1.

(2*S*)-1-((2*R*)-3-(Acetylthio)-2-methylpropanoyl)pyrrolidine-2-carboxylic acid (**12**)

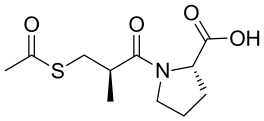

A solution (2*R*)-3-(acetylthio)-2-methylpropanoic acid (**10**, 162 mg, 1.0 mmol) in CH_2Cl_2 (3 mL) was cooled to 0 °C after which $SOCl_2$ (73 µL, 1.0 mmol) was added slowly. The reaction was slowly warmed to r.t.; then stirred overnight. Volatiles were removed *in vacuo* and the residual oil was re-dissolved in CH_2Cl_2 and subsequently evaporated-off to remove residual $SOCl_2$. The crude acetyl chloride (**11**) was dissolved in CH_2Cl_2 (5 mL) where after L-Pro-OH (115 mg, 1.0 mmol) was added, followed by the addition of Et_3N (418 µL, 3.0 mmol). The reaction was stirred at r.t. for 16 hours. The reaction was quenched by the addition of 1M HCl (5 mL). After extraction and separation of the layers, the organic layer was dried over Na_2SO_4, filtered and concentrated *in vacuo*. The crude product was purified by column chromatography (CH_2Cl_2/MeOH/AcOH, 9:1:0.05) to yield **12** as a waxy solid (123 mg, 47%). R_F = 0.1 (cHex/EtOAc, 1:1). ^{1}H NMR (400 MHz, $CDCl_3$) (Signals for major conformational isomer are reported) δ = 4.57-4.64 (m, 1H), 3.72-3.80 (m, 1H), 3.49 (td, *J* = 9.5, 7.0 Hz, 1H), 3.10-3.18 (m, 1H), 2.93-3.00 (m, 1H) 2.88 (dq, *J* = 14.0, 6.5 Hz, 1H) 2.43-2.51 (m, 1H), 2.35 (s, 3H), 1.95-2.09 (m, 3H), 1.24 (d, *J* = 6.5 Hz, 3H) ppm. LRMS calcd. for $C_{11}H_{16}NO_4S$, M-H = 258.08, mass found; 258.1.

(2*S*)-1-((2*R*)-3-Mercapto-2-methylpropanoyl)pyrrolidine-2-carboxylic acid (*epi*-L-captopril, **3**)

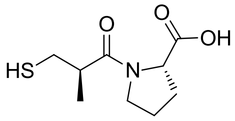

(2*S*)-1-((2*R*)-3-(Acetylthio)-2-methylpropanoyl)pyrrolidine-2-carboxylic acid (**12**, 123 mg, 0.47 mmol) was dissolved in 1M HCl (5 mL) and subsequently heated to 90 °C for 4 hours. After completion of the reaction, the reaction was cooled to r.t. and the product was extracted with CH_2Cl_2 (3 x 10 mL). The combined organic layers were dried over Na_2SO_4, filtered and concentrated *in vacuo*. The crude product was purified by column chromatography (CH_2Cl_2/MeOH/AcOH, 9:1:0.05) to yield **3** as colorless oil (36 mg, 35%). R_F = 0.3 (CH_2Cl_2/MeOH/AcOH, 9:1:0.05). ^{1}H NMR (400 MHz, $CDCl_3$) (Signals for major conformational isomer are reported) δ = 4.63 (dd, *J* = 8.0, 2.5 Hz, 1H), 3.81-3.88 (m, 1H), 3.46-3.57 (m, 1H), 2.82-3.00 (m, 2H), 2.41-2.53 (m, 2H), 1.98-2.17 (m, 3H), 1.65 (dd, *J* = 10.0, 7.5 Hz, 1H), 1.22 (d, *J* = 6.5 Hz, 3H) ppm. LRMS calcd. for $C_9H_{14}NO_3S$, M-H = 216.07, mass found; 216.1.

(2*R*)-Methyl 1-((2*R*)-3-(acetylthio)-2-methylpropanoyl)pyrrolidine-2-carboxylate (**13**)

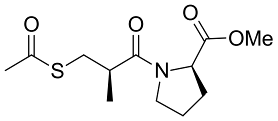

A solution (2*R*)-3-(acetylthio)-2-methylpropanoic acid (**10**, 162 mg, 1.0 mmol) in CH_2Cl_2 (3 mL) was cooled to 0 °C, afterwhich $SOCl_2$ (73 µL, 1.0 mmol) was added slowly. The reaction was slowly warmed to r.t., then stirred overnight. Volatiles were removed *in vacuo* and the remaining oil was re-dissolved in CH_2Cl_2 and subsequently evaporated to remove $SOCl_2$. The crude acetyl chloride (**11**) was dissolved in CH_2Cl_2 (5 mL), then D-Pro-OMe.HCl (165 g, 1.0 mmol) was added, followed by tEt_3N (418 µL, 3.0 mmol). The reaction was stirred at r.t. for 16 hours, then quenched by addition of 1M HCl (5 mL). After extraction and separation of layers, the organic layer was dried over Na_2SO_4, filtered and concentrated *in vacuo*. The crude product was purified by column chromatography (cHex/EtOAc, 2:1→ 1:1) to yield **13** as a waxy solid (175 mg, 64%). R_F = 0.35 (cHex/EtOAc, 1:1). ^1H NMR (400 MHz, $CDCl_3$) (Signals for major conformational isomer are reported) δ = 4.52 (dd, *J* = 8.5, 4.0 Hz, 1H), 3.72 (s, 3H), 3.62 (t, *J* = 6.5 Hz, 2H), 3.07-3.14 (m, 1H), 2.93-3.00 (m, 1H) 2.80 (sxt, *J* = 7.0 Hz, 1H), 2.33 (s, 3H) 2.19-2.27 (m, 1H), 1.95-2.13 (m, 3H) 1.24 (d, *J* = 7.0 Hz, 3H) ppm. LRMS calcd. for $C_{12}H_{19}NO_4S$, M+H = 274.09, mass not found.

(2*R*)-1-((2*R*)-3-Mercapto-2-methylpropanoyl)pyrrolidine-2-carboxylic acid (*epi*-D-captopril, **4**)

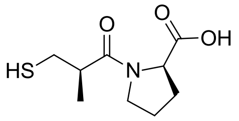

(2*R*)-1-((2*R*)-3-(Acetylthio)-2-methylpropanoyl)pyrrolidine-2-carboxylic acid (**13**, 175 mg, 0.64 mmol) was dissolved in 1M HCl (5 mL), then heated to 90 °C for 4 hours. After completion of the reaction, the reaction was cooled to r.t. and the product was extracted with CH_2Cl_2 (3 x 10 mL). The combined organic layers were dried over Na_2SO_4, filtered and concentrated *in vacuo*. The crude product was purified by column chromatography (CH_2Cl_2/MeOH/AcOH, 9:1:0.05) to yield **4** as a colorless oil (42 mg, 30%). R_F = 0.3 (CH_2Cl_2/MeOH/AcOH, 9:1:0.05). ^1H NMR (400 MHz, $CDCl_3$) (Signals for major conformational isomer are reported) δ = 4.69 (dd, *J* = 8.0, 2.5 Hz, 1H), 3.57-3.71 (m, 2H), 2.79-2.97 (m, 2H), 2.49-2.57 (m, 2H), 1.95-2.13 (m, 3H), 1.56 (dd, *J* = 9.0, 8.5 Hz, 1H), 1.24 (d, *J* = 6.5 Hz, 3H) ppm. LRMS calcd. for $C_9H_{14}NO_3S$, M-H = 216.07, mass found; 216.1.

2-(Acetylthio)benzoic acid (**15**) (7)

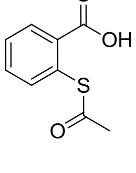

15 was prepared according to the literature procedure (5): A mixture containing thiosalicilic acid **14** (1.0 g, 6.49 mmol), acetic acid anhydride (0.73 mL, 7.74 mmol) and acetic acid (3 mL) was heated to

100 °C under an N_2-atmosphere. After 2 hours the reaction was cooled to r.t. and subsequently poured in a cooled solution (0 °C) of 1M HCl (20 mL). The resultant solution was left standing at (0 °C) for 30 min. The resulting white solid was filtered-off and washed with H_2O (20 mL). The product 15 was obtained as a white solid (720 mg, 56%) after drying under high vacuum overnight. ^{1}H NMR (400 MHz, $CDCl_3$) δ = 8.10 (d, *J* = 8.0 Hz, 1H), 7.56-7.62 (m, 2H), 7.55-7.50 (m, 1H), 2.46 (s, 3H) ppm (in accordance with the literature values) (7).

(*S*)-Methyl 1-(2-(acetylthio)benzoyl)pyrrolidine-2-carboxylate (**16**) (5)

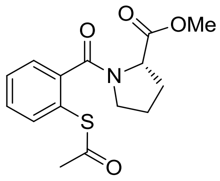

16 was prepared according to the literature procedure (5): A solution containing 2-(acetylthio)benzoic acid (**15**, 196 mg, 1.0 mmol) in CH_2Cl_2 (10 mL) was cooled to 0 °C after which L-Pro-OMe.HCl (165 mg, 1.0 mmol), DMAP (121 mg, 1.0 mmol) and DCC (216 mg, 1.05 mmol) were added to the solution. The reaction was stirred at 0 °C for 30 min followed by r.t. overnight. The reaction was quenched by the addition of 1M HCl (10 mL) and the layers were separated. The organic layer was dried over Na_2SO_4, filtered and concentrated. The crude product was purified by column chromatography (cHex/EtOAc, 9:1 → 1:1) to give **16** as a white solid (187 mg, 75%). R_F = 0.35 (cHex/EtOAc, 9:1) ^{1}H NMR (400 MHz, $CDCl_3$) δ = 7.40-7.55 (m, 4H), 4.65 (dd, *J* = 8.5, 5.0 Hz, 1H), 3.79 (s, 3H), 3.36-3.44 (m, 1H), 3.33-3.27 (m, 1H), 2.44 (s, 3H), 2.24-2.35 (m, 1H) 2.09-2.03 (m, 3H), 1.79-1.89 (m, 1H) ppm. LRMS calcd. for $C_{15}H_{16}NO_4S$, M+H = 307.09, mass found; M+H = 308.1

(*S*)-1-(2-Mercaptobenzoyl)pyrrolidine-2-carboxylic acid (*S*-MBP, **17**) (8)

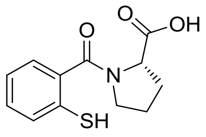

To a solution of (*S*)-methyl 1-(2-(acetylthio)benzoyl)pyrrolidine-2-carboxylate (**16**, 187 mg, 0.61 mmol) in dioxane (5 mL) was added 1M HCl (5 mL). The reaction mixture was heated to 90 °C for 3 hours. After complete conversion the reaction was cooled to r.t. and extracted with CH_2Cl_2 (3 x 10 mL). The combined organic layers were dried over Na_2SO_4, filtered and concentrated *in vacuo*. The crude product was purified by column chromatography (CH_2Cl_2/MeOH/AcOH, 9:1:0.05) to yield the **17** as a colorless oil (105 mg, 68%). R_F = 0.30 (CH_2Cl_2/MeOH/AcOH, 9:1:0.05). ^{1}H NMR (400 MHz, $CDCl_3$) δ = 7.15-7.40 (m, 4H), 4.78 (dd, *J* = 8.5, 4.5 Hz, 1H), 4.07 (br. s., 1H), 3.40-3.51 (m, 1H), 3.29-3.39 (m, 1H), 2.34-2.45 (m, 1H), 2.18-2.32 (m, 1H), 1.89-2.09 (m, 2H) ppm. LRMS calcd. for $C_{12}H_{13}NO_3S$, M-H = 250.05, mass found; M-H = 250.1

(*R*)-Methyl 1-(2-(acetylthio)benzoyl)pyrrolidine-2-carboxylate (**18**)

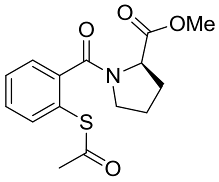

18 was prepared according to a literature procedure (5): A solution containing 2-(acetylthio)benzoic acid (15, 196 mg, 1.0 mmol) in CH_2Cl_2 (10 mL) was cooled to 0 °C after which D-Pro-OMe.HCl (165 mg, 1.0 mmol), DMAP (121 mg, 1.0 mmol) and DCC (216 mg, 1.05 mmol) were added. The reaction was stirred at 0 °C for 30 min then at r.t. overnight. The reaction was quenched by the addition of 1M HCl (10 mL) and the layers were separated. The organic layer was dried over Na_2SO_4, filtered and concentrated. The crude product was purified by column chromatography (cHex/EtOAc, 9:1 → 1:1) to give **18** as a white solid (219 mg, 71%). R_F = 0.25 (cHex/EtOAc, 9:1) ^{1}H NMR (400 MHz, $CDCl_3$) δ = 7.39-7.54 (m, 4H), 4.65 (dd, *J* = 8.5, 5.0 Hz, 1H), 3.79 (s, 3H), 3.43-3.37 (m, 1H), 3.26-3.34 (m, 1H), 2.44 (s, 3H), 2.24-2.35 (m, 1H) 1.90-2.10 (m, 3H) 1.80 - 1.89 (m, 1H) ppm.

(*R*)-1-(2-Mercaptobenzoyl)pyrrolidine-2-carboxylic acid (*R*-MBP, **19**)

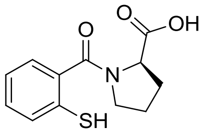

To a solution of (*R*)-methyl 1-(2-(acetylthio)benzoyl)pyrrolidine-2-carboxylate (**18**, 219 mg, 0.71 mmol) in dioxane (5 mL) was added 1M HCl (5 mL). The reaction mixture was heated to 90 °C for 3 hours. After the reaction was complete by TLC analysis, it was cooled to r.t. and extracted with CH_2Cl_2 (3 x 10 mL). The combined organic layers were dried over Na_2SO_4, filtered and concentrated *in vacuo*. The crude product was purified by column chromatography (CH_2Cl_2/MeOH/AcOH, 9:1:0.05) to yield the **19** as a colorless oil (123 mg, 81%). R_F = 0.30 (CH_2Cl_2/MeOH/AcOH, 9:1:0.05). ^{1}H NMR (400 MHz, $CDCl_3$) δ = 7.16-7.42 (m, 4H), 5.53 (br. s., 1H), 4.79 (dd, *J* = 8.5, 4.5 Hz, 1H), 4.06 (br. s., 1H), 3.23-3.50 (m, 2H), 2.34-2.44 (m, 1H), 2.16-2.33 (m, 1H), 1.84-2.09 (m, 2H) ppm. LRMS calcd. for $C_{12}H_{13}NO_3S$, M-H = 250.05, mass found; M-H = 249.9

References

1. **Wallace AC, Laskowski RA, Thornton JM.** 1995. LIGPLOT: a program to generate schematic diagrams of protein-ligand interactions. Protein Eng **8:**127-134.
2. **Skiles JW, Suh JT, Williams BE, Menard PR, Barton JN, Loev B, Jones H, Neiss ES, Schwab A.** 1986. Angiotensin-converting enzyme inhibitors: new orally active 1,4-thiazepine-2,5-diones, 1,4-thiazine-2,5-diones, and 1,4-benzothiazepine-2,5-diones possessing antihypertensive activity. J Med Chem **29:**784-796.
3. **Heinz U, Bauer R, Wommer S, Meyer-Klaucke W, Papamichaels C, Bateson J, Adolph HW.** 2003. Coordination geometries of metal ions in d- or l-captopril-inhibited metallo-beta-lactamases. J Biol Chem **278:**20659-20666.
4. **Kim BH, Lee HB, Hwang JK, Kim YG.** 2005. Asymmetric induction in the conjugate addition of thioacetic acid to methacrylamides with chiral auxiliaries. Tetrahedron: Asymmetry **16:**1215-1220.
5. **Menard PR, Suh JT, Jones H, Loev B, Neiss ES, Wilde J, Schwab A, Mann WS.** 1985. Angiotensin converting enzyme inhibitors. (Mercaptoaroyl)amino acids. J Med Chem **28:**328-332.
6. **Gottlieb HE, Kotlyar V, Nudelman A.** 1997. NMR Chemical Shifts of Common Laboratory Solvents as Trace Impurities. J Org Chem **62:**7512-7515.
7. **Kikionis S, McKee V, Markopoulos J, Igglessi-Markopoulou O.** 2008. A prominent C-acylation–cyclisation synthetic sequence and X-ray structure elucidation of benzothiopyranone derivatives. Tetrahedron **64:**5454-5458.
8. **Nacci V, Garofalo A, Anzini M, Campiani G.** 1988. Polycondensed heterocycles. III. Synthesis of 5,11-dioxo-1,2,3,1 1a-tetrahydro-5H,11H- and 5-oxo-2,3,11,1 1a-tetrahydro-1H,5H-pyrrolo[2,1-c][1,4]benzothiazepine. J Heterocyclic Chem **25:**1007-1013.
